# Applications of machine learning for computer-aided diagnosis of Parkinson’s disease: progress and benchmark case study

**DOI:** 10.1007/s10462-025-11347-y

**Published:** 2025-08-29

**Authors:** Juntao Zhang, Yiming Zhang, Ying Weng, Akram A. Hosseini, Boding Wang, Tom Dening, Weinyu Fan, Weizhong Xiao

**Affiliations:** 1 School of Computer Science, University of Nottingham Ningbo China, Ningbo, 315100 China; 2 School of Computer Science, University of Nottingham, Nottingham, NG8 1BB UK; 3 Department of Academic Neurology, Queen’s Medical Centre, Nottingham University Hospitals NHS Trust, Nottingham, NG7 2UH UK; 4 Neurology Department, Ningbo No.2 Hospital, Ningbo, 315010 China; 5 School of Medicine, University of Nottingham, Nottingham, NG7 2UH UK; 6 Neurology Department, Peking University Third Hospital, Beijing, 100191 China

**Keywords:** Parkinson’s disease (PD), Machine learning (ML), Deep learning (DL), Computer-aided diagnosis (CAD), Case study

## Abstract

Machine learning (ML) has emerged as a vital tool for the diagnosis of Parkinson’s Disease (PD). This study presents a comprehensive review on the applications of ML for computer-aided diagnosis (CAD) of PD. We conducted a comprehensive review by searching articles published from 2010 till 2024. The risk of bias is assessed using the PROBAST checklist. Case studies are also provided. This review includes 117 articles with six categories: neuroimaging data (20.5%); voice data (40.2%); handwriting data (12.0%); gait data (14.5%); EEG data (8.5%); and other data (4.3%). According to the PROBAST checklist, only 28 articles (23.9%) have a low risk of bias. A benchmark case study is conducted for five different data modalities. We also discuss current limitations and future directions of applying ML to the diagnosis of PD. This review reduces the gap between Artificial Intelligence (AI) and PD medical professionals and provides helpful information for future research.

## Introduction

Parkinson’s disease (PD) is the second-leading progressive neurodegenerative disorder after Alzheimer’s disease (AD) and is characterised by numerous motor and non-motor features (Jankovic 2008). Its incidence tends to increase, especially beyond the age of 60 years. PD is diagnosed based on the patient’s medical history and clinical criteria, and there is no definitive test or laboratory test for PD diagnosis (Jankovic 2008). It is a challenge for the medical specialist to correctly differentiate PD from other pathologies when the signs and symptoms of the patients overlap with other Parkinsonian syndromes (Trifonova et al. 2020). Hence, it is important to assess whether applying Computer-aided diagnosis (CAD) will help the medical specialist to aid the diagnosis of PD. Artificial Intelligence (AI) has been found helpful in healthcare, and it has been utilised for disease detection, diagnosis, treatment, and prognosis evaluation (Jiang et al. 2017). In the past 15 years, many AI methods have been applied in the field of CAD of PD. In particular, deep learning (DL) has become more attractive in the last decade than conventional Machine learning (ML), as it can discover and learn more hidden patterns from healthcare data (LeCun et al. 2015). For example, ML and DL-based methods have been applied as computer-assisted techniques on the diagnosis of brain diseases using neuroimaging data (Li et al. 2014), including the diagnosis of AD (Liu et al. 2014) and PD. Moreover, PD diagnosis using ML involves high data complexity due to the variety of data modalities, such as neuroimaging, gait, voice, and handwriting (Cano 2013). These datasets are often high-dimensional and may contain noise, making preprocessing and analysis more challenging (Khan et al. 2023, 2024; Perumal et al. 2025). To comprehensively examine the research progress over the past 15 years and provide meaningful guidance on the application of ML in the medical domain, we conduct a systematic review of ML-based computer-aided diagnosis for PD. Unlike previous review papers (Zhang 2022; Sigcha et al. 2023; Wang et al. 2024), which neither focused on a limited number of data modalities nor lacked practical benchmarking efforts, our work introduces case studies that directly address the gaps in the field. To ensure methodological rigor, we select five of the most commonly used data modalities and use a public dataset. Additionally, to support transparency and reproducibility, we have released the code implementations of all benchmark case studies via GitHub: https://github.com/yiming95/PD_ML_benchmark.

### Search strategy

**Fig. 1:**
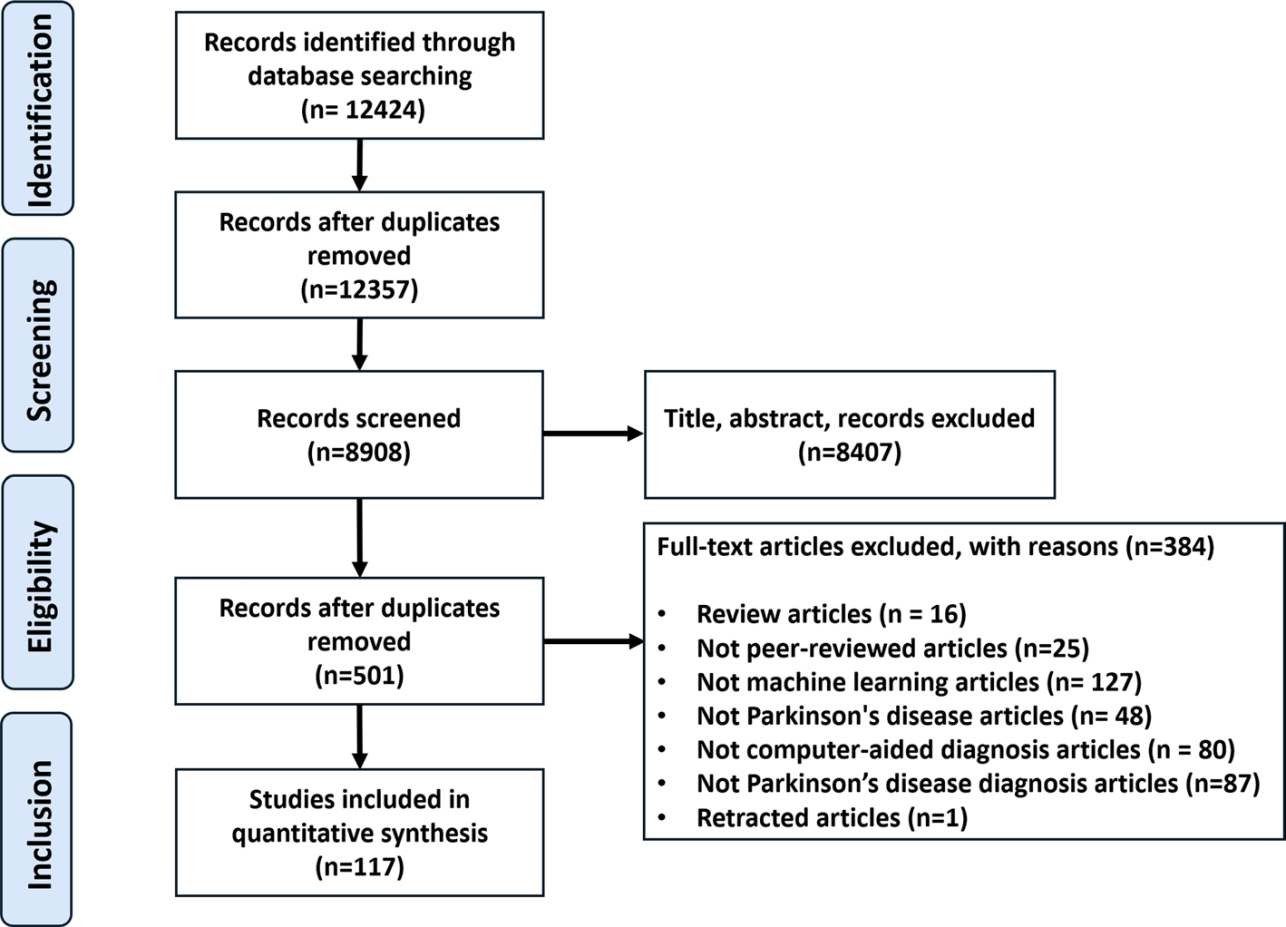
PRISMA Flow chart. The study selection process shows the number of the literature identified, screened, assessed, and included in this systematic review

We perform this systematic review of literature on PD diagnosis using ML techniques following the Preferred Reporting Items for Systematic Review and Meta-Analyses (PRISMA) statement (Moher et al. 2009). Four electronic databases (1) IEEE Xplore, (2) Association for Computing Machinery (ACM), (3) Springer, and (4) Science Direct are searched for relevant publications from 2010 to 2024. Google Scholar and PubMed are searched between these dates for potentially relevant studies as well. We use several keywords as search queries, including “Parkinson’s disease”, “PD”, "Diagnosis”, “Diagnostics”, “Computer-Aided Diagnosis”, “Deep learning”, “Machine learning”, and “Artificial Intelligence”. The PRISMA flowchart is shown in Fig. 1.

The review aimed to identify the publications on PD diagnosis using ML. All included articles focus on the topic of PD diagnosis using ML. Besides, only publications in English are included. The publications focusing on the treatment or prognostic evaluation of PD, or those only using image analysis or signal analysis methods, are excluded. Review papers and non-peer-reviewed papers are also excluded. Publications were first screened for eligibility by Title and Abstract. Potentially eligible studies were then assessed and evaluated in full text. We then analyze and extract data from the screened articles. Data extracted from the full-text articles include (1) Author, (2) Published year, (3) Objective, (4) Data modality, (5) Dataset, (6) Number of subjects, (7) ML algorithms applied, (8) Validation, (9) Evaluation metrics. The results section analyzes five different data modalities from the main public datasets that have been used by ML researchers, including neuroimaging data, voice data, handwriting data, gait data, and electroencephalogram (EEG) data. The meta-analysis is not performed due to the heterogeneity of the included studies.

### Contributions

This interdisciplinary systematic review quantifies and analyzes the last 15 years’ publications on the diagnosis of PD using ML techniques. By conducting a benchmark case study on five commonly used modalities, including MRI, gait, voice, EEG, and handwriting, we find the issues in this field, which are that the reported results are hard to reproduce and lack interpretability analysis. Furthermore, this systematic review aims to summarize the current trends of how ML techniques are applied in the early diagnosis of PD. Besides, it also aims to identify the current limitations and challenges of applying ML in the diagnosis of PD and propose a few promising future directions. Compared to previous works, this article encompasses the broadest range of literature from 2010 to 2024 and includes the largest number of modalities. Additionally, no prior works have conducted a detailed case study experiment to test the reproducibility of results across multiple modalities. The contributions of this paper can be summarized into:

- We conduct a systematic review on ML-based CAD for PD applications published from 2010 to 2024. Specifically, we analyze the data modalities, dataset, ML algorithm, and model performance for each study.
- We conduct a comprehensive case study on five data modalities.
- The paper also discusses the current limitations and future directions of applying ML in PD diagnosis.

The rest of the paper is organized as follows. Section 2 summarizes the ML-based PD diagnosis applications and introduces the datasets and evaluation metrics. Section 3 shows the results of the risk of bias assessment. Section 4 shows the details of the case study. Section 5 provides the discussion, including a summary of the findings, current challenges, and future research directions. Section 6 summarizes the paper.

## Applications of ML-based PD diagnosis

**Fig. 2:**
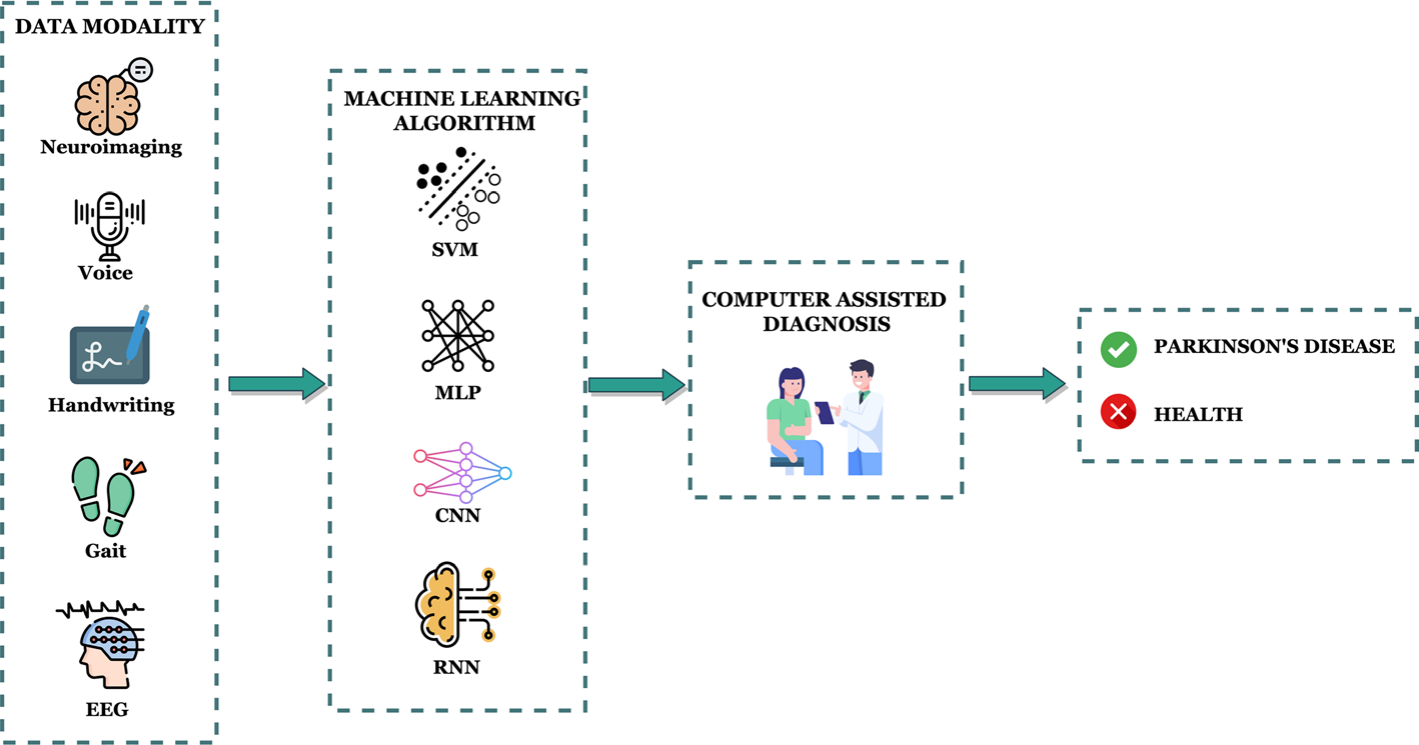
Pipeline for the general ML-based computer-assisted PD diagnosis

Based on our search and study selection process, we first identified 12424 articles from IEEE Xplore, ACM, Springer, and Science Direct. Additional articles are also included from Google Scholar and PubMed. After removing duplicates, 8908 articles are then screened for eligibility. After screening the article’s Title and Abstract, we excluded 8407 articles, leaving 501 articles for full-text examination. Finally, we include 117 articles for data extraction. A general procedure pipeline for PD diagnosis using ML is shown in Fig 2. Table 1 shows all the included studies.

### Neuroimaging data

**Fig. 3:**
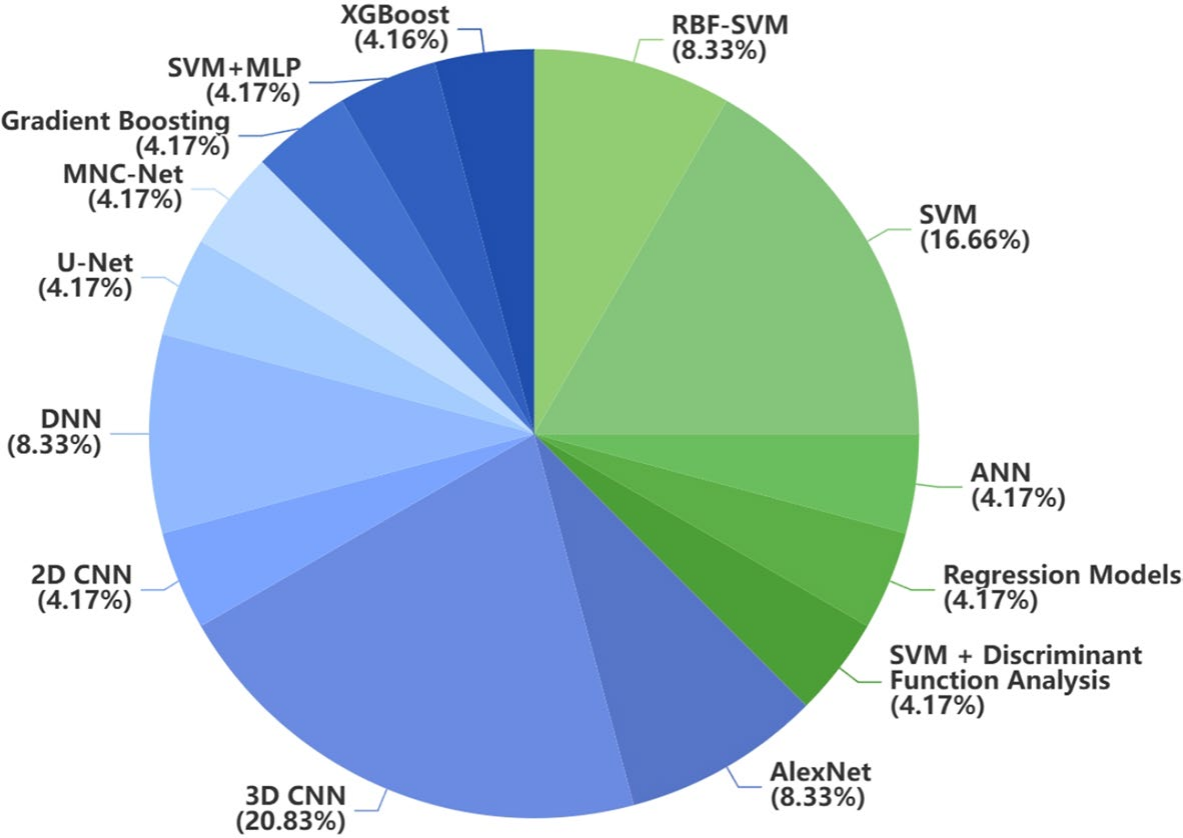
Distribution of traditional ML method and DL method in neuroimaging data. Blue represents DL and green represents traditional ML

**Fig. 4:**
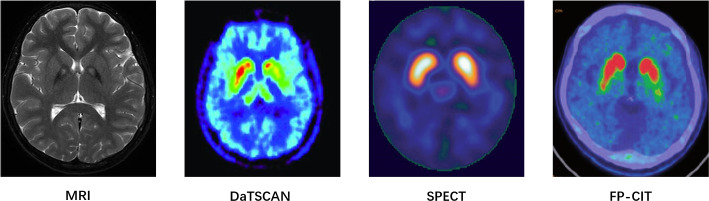
The samples of neuroimaging data

Neuroimaging is a branch of medical imaging that applies radiological and other techniques to images of the nervous system (Rastogi et al. 2025; Kujur et al. 2022; Alhussen et al. 2025). With the increasing availability of large-scale neuroimaging datasets and advancements in ML and DL, neuroimaging has played an important role in the early detection, classification, computer-aided diagnosis, and monitoring of various neurological disorders (Goceri 2024, 2025; Nakach et al. 2024). Many studies have applied neuroimaging for the early diagnosis of PD using ML techniques. In this review, we include 18 articles using neuroimaging data. Among them, 12.5% of the articles (3/24) used SPECT data, 20.8% of the articles used (5/24) DTI data, 62.5% of the articles used (15/24) MRI data, and 4.2% (1/24) of the articles used Positron Emission Tomography (PET) imaging data. In terms of ML models, the most commonly used models are Support Vector Machine (SVM), Convolutional Neural Network (CNN), and 3D CNN. Besides, some data-preprocessing techniques are used to minimise the noise of the image. Data augmentation techniques, such as Generative Adversarial Network (GAN), may be used to increase the number of samples. For validation, various methods are used, including 10-fold cross-validation; train, validation, and test split validation; and train and test split validation. 45.8% (11/24) of the articles reported an accuracy of over 90%. There are also some problems with applying neuroimaging data to PD diagnosis. For example, some comparisons between previous studies are unfair as they applied different experimental datasets or the same dataset with different subjects. Besides, some studies only applied the train and test split validation, which is unsuitable because the dataset size is small. Fig 3 presents the distribution of traditional ML and DL approaches employed in neuroimaging-based studies. Since most neuroimaging data are saved in the form of medical imaging (Fig 4), the application of DL in neuroimaging datasets is more widespread than that of traditional ML.

### Voice data

Analysis of voice or speech characteristics could contribute to PD diagnosis and detection, especially as recent research has shown that voice impairment is the commonest underlying symptom in many PD patients (Karan et al. 2020). In PD diagnosis based on voice data, 57.1% of the articles (28/47) used the dataset collected from the University of Oxford (Tsanas et al. 2012). However, the dataset size is too small (only contains 31 participants, and the data distribution is unbalanced (23 PD patients and 8 healthy controls). These disadvantages cause the model to have a weak generalisation. For the model evaluation, 55.3% of the articles (26/47) used cross-validation, where 10-fold cross-validation was the most common method (18/47). Unfortunately, 14.9% of the articles (7/47) did not provide a detailed evaluation method. In addition, there was no uniform standard for splitting datasets. Some of the same speech samples often appeared in both the training set and testing set, which led to overly optimistic performance results.

**Fig. 5:**
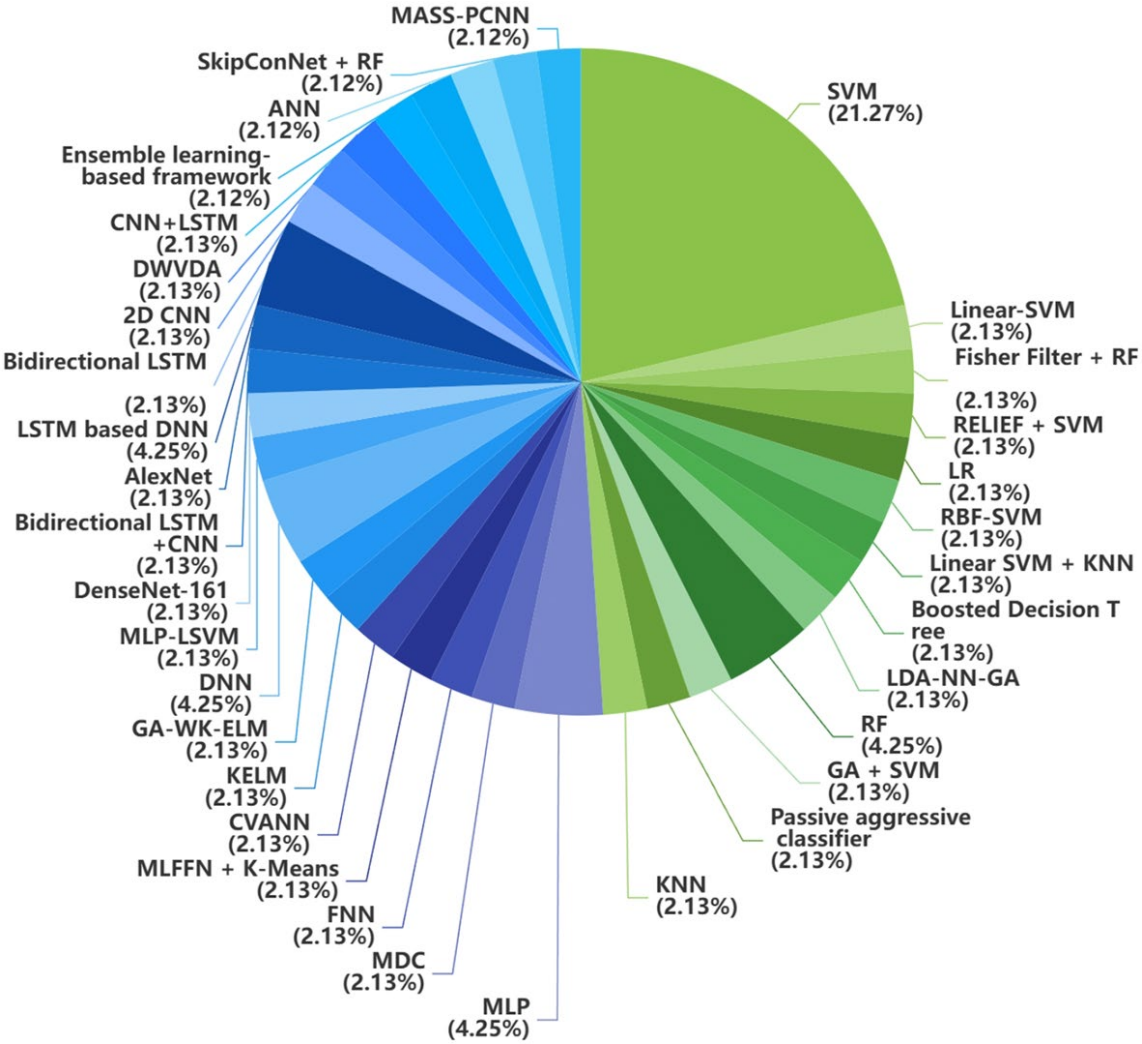
Distribution of traditional ML method and DL method in voice data. Blue represents DL and green represents traditional ML

Overall, voice data is the most widely used data modality but has limited potential to apply in the real world due to different languages, accents, and uncontrollable ambient sounds. One model may perform well on one specific dataset, but poorly on another. Many articles simply quoted the performance data from other studies rather than undertaking their own evaluation. Fig 5 presents the distribution of traditional ML and DL approaches employed in voice-based studies.

### Handwriting data

**Fig. 6:**
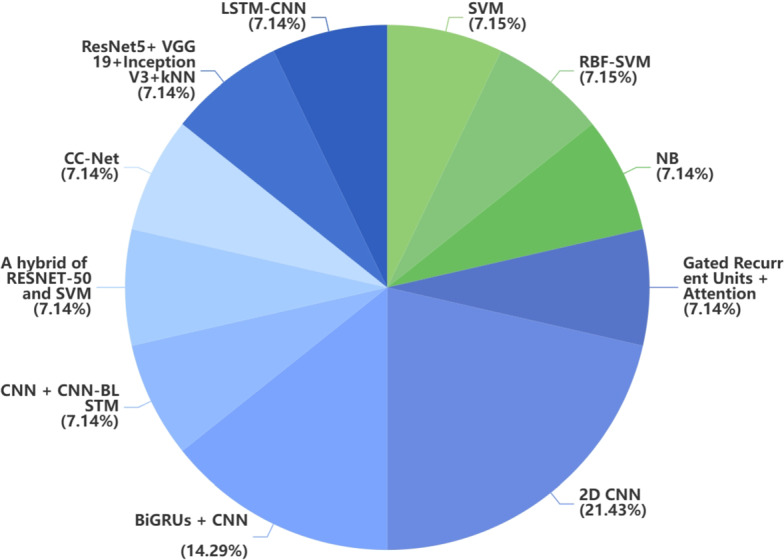
Distribution of traditional ML method and DL method in handwriting data. Blue represents DL and green represents traditional ML

**Fig. 7:**
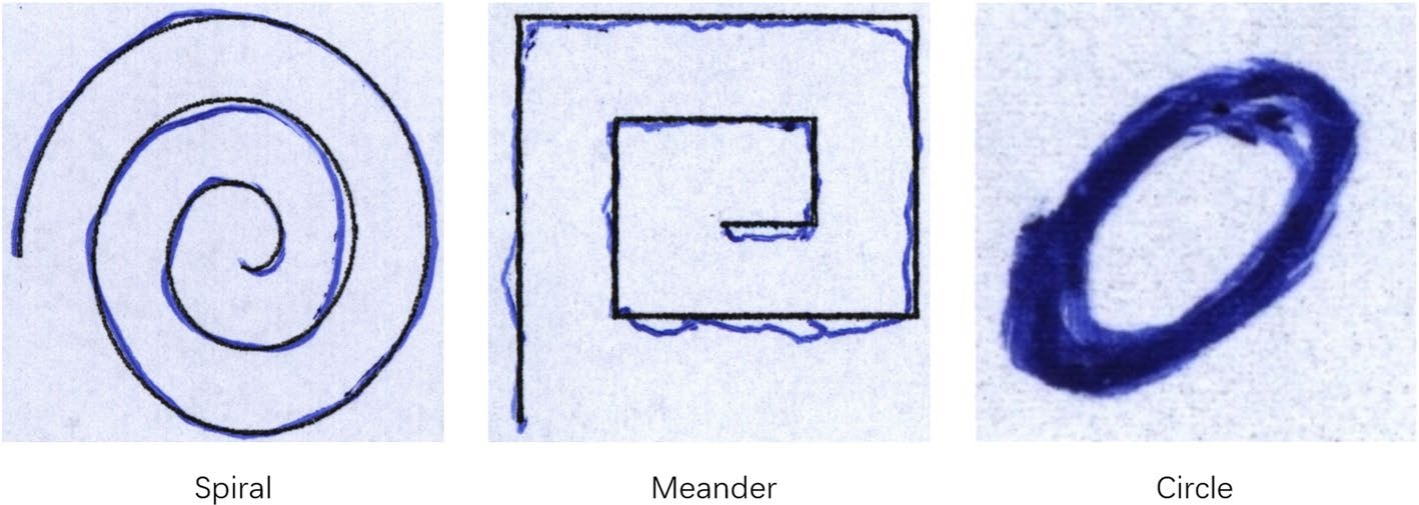
The samples of handwriting data

Handwriting requires motor control and specific neuromuscular coordination. Handwriting abnormalities are a common early motor symptom of PD and, therefore, of potential value for diagnosis. The number of participants included in studies of handwriting-based PD diagnosis is relatively small. 14.3% (2/14) of the articles used a study population of more than 200, while 85.7% (12/14) of the articles included fewer than 200 patients, where SVM, CNN, and RNN were the most commonly used ML models. Regarding validation, 71.4% (10/14) of the articles applied k-fold cross-validation, and only 28.6% (4/14) of the articles used the train and test split validation mechanism. 57.1% (8/14) of the articles reported a diagnostic accuracy of over 90%, and 92.9% (13/14) of the articles reported an accuracy of over 80%. Fig 6 presents the distribution of traditional ML and DL approaches employed in handwriting-based studies. Since most handwriting datasets are saved in the form of pictures(Fig 7), the application of DL in handwriting datasets is more widespread than that of traditional ML.

### Gait data

Gait disorder is one of the most incapacitating motor symptoms in PD and a challenge for the medical specialist to evaluate. In PD diagnosis based on gait data, 64.7% of the articles (11/17) used the dataset from Physionet. This dataset contains 166 subjects (93 PD patients and 73 healthy controls (HC)). 58.8% of the articles (10/17) used cross-validation, where 10-fold cross-validation was the most common method (9/17).

**Fig. 8:**
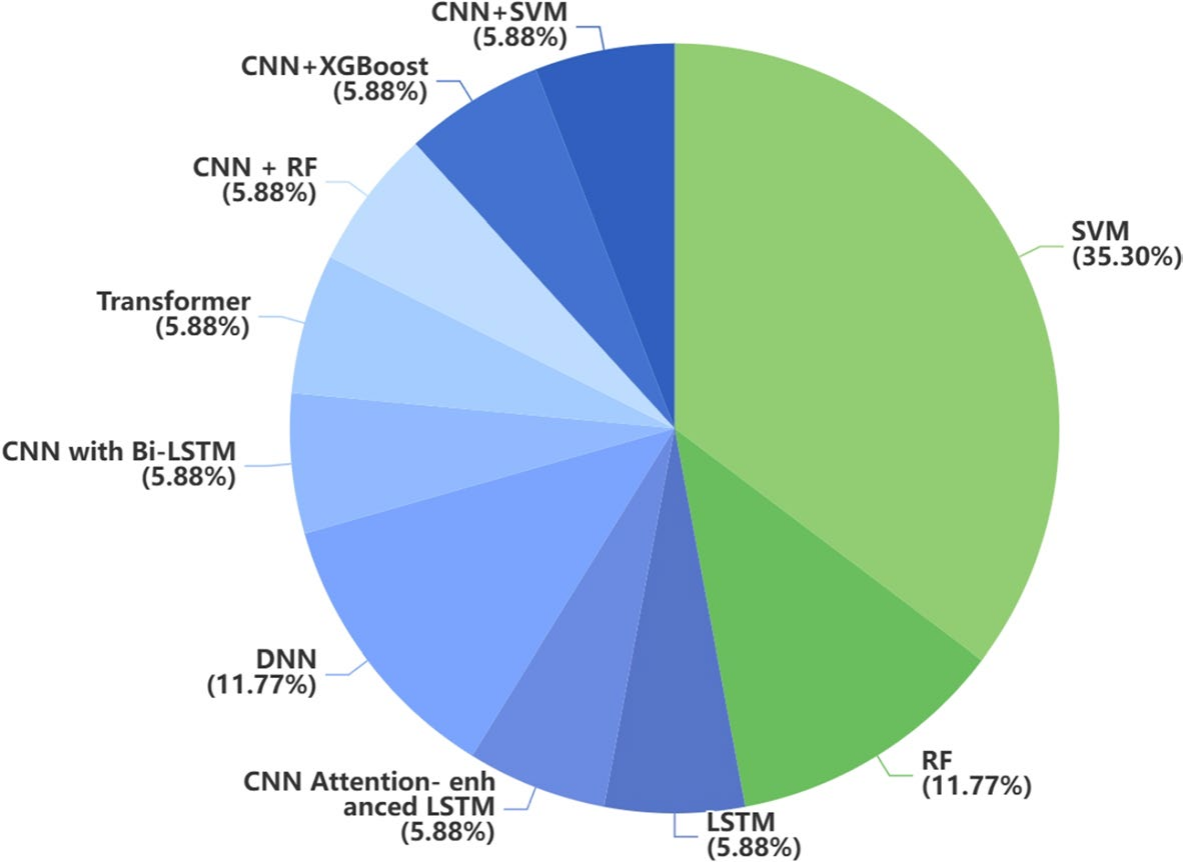
Distribution of traditional ML method and DL method in gait data. Blue represents DL and green represents traditional ML

The data in the dataset needs to be segmented according to the gait cycle; otherwise, some specific data samples may be located at the intersection part of the probability density functions for two classes. Moreover, extracting the features of the left and right gait separately may result in better performance. The gait data-based model is highly generalizable since walking posture is similar for people from different countries. Fig 8 presents the distribution of traditional ML and DL approaches employed in gait-based studies.

### EEG data

**Fig. 9:**
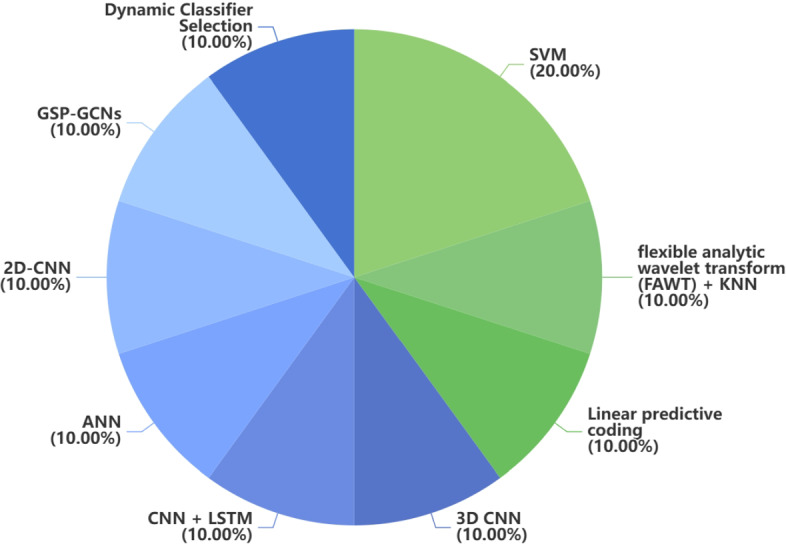
Distribution of traditional ML method and DL method in EEG data. Blue represents DL and green represents traditional ML

EEG involves recording brain signals from the scalp’s surface. As PD is related to brain abnormalities, EEG signals can be applied to assist in PD diagnosis. Ten articles are included in this review. 60.0% (6/10) of the articles included fewer than 50 participants. 30.0% (3/10) of the articles used CNN-based models. 40.0% (4/10) articles applied 10-fold cross-validation, and 30.0% (3/10) articles applied to train and test split validation. Fig 9 presents the distribution of traditional ML and DL approaches employed in EEG-based studies.

### Other data

**Fig. 10:**
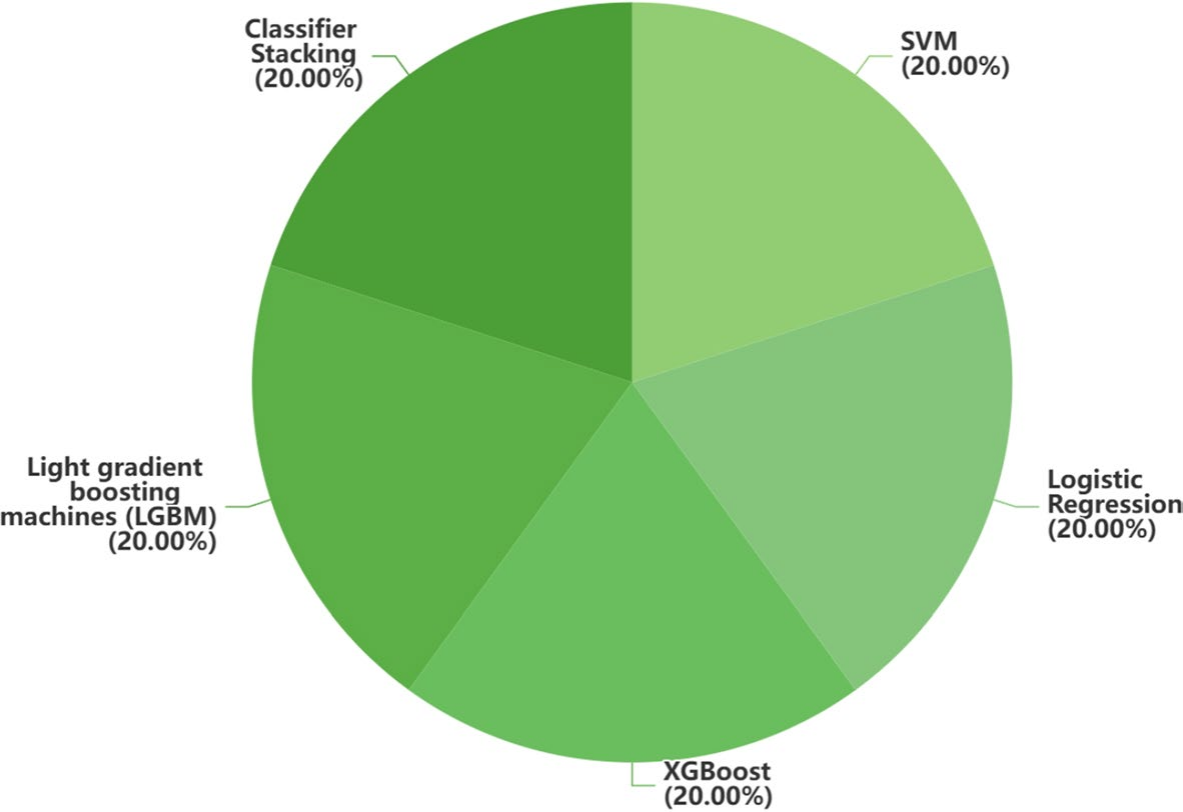
distribution of traditional ML method and DL method in other data. Blue represents DL and green represents traditional ML

Besides these data modalities, this review also includes five research studies that used other data modalities, such as gene and urine biomarkers. Out of these, 60% (3/5) of articles used 10-fold cross-validation. Fig 10 presents the distribution of traditional ML and DL approaches employed in other data-based studies. Due to the limited computer science background of most authors who collected these new datasets, and the majority of datasets were recorded in the form of indicators or textual descriptions, traditional ML methods were chosen over DL approaches.

### Datasets

We briefly summarize the five commonly used public datasets for ML-based PD diagnosis.

***PPMI*** Parkinson’s Progression Markers Initiative (PPMI) dataset was sponsored by the Michael J. Fox Foundation (MJFF). It is a dataset used for PD diagnosis with neuroimaging data modality. The study contains imaging, clinical, and biological data on PD patients and the HC group. It is designed to define and discover biomarkers of PD progression.

***PC-GITA*** PC-GITA dataset, also called the new Spanish speech corpus dataset, is the first dataset that provides speech recordings in Spanish (Orozco-Arroyave et al. 2014). It is a dataset used for PD diagnosis with voice data modality. This dataset contains speech recordings of 50 PD patients and 50 HC subjects, where all subjects are native Spanish speakers. The speech recordings were collected following a designed protocol, and the corpus dataset includes several tasks such as sustained phonations of the vowels and diadochokinetic evaluation.

***HandPD*** The HandPD dataset is used for PD diagnosis with handwriting data and contains 55 subjects with 37 PD patients, and 18 HC subjects. Each subject was asked to complete the handwriting clinical exam, such as drawing spirals and circles (Pereira et al. 2015). As some subjects did not complete all of the exam tasks, the entire dataset comprises 373 images.

***PaHaW*** Parkinson’s Disease Handwriting (PaHaW) dataset consists of 75 subjects with 37 PD patients and 38 HC subjects (Drotár et al. 2016). It is a dataset used for PD diagnosis with handwriting data. The tasks include drawing an Archimedean spiral, repetitively writing orthographically simple syllables and words, and writing a sentence.

***Physionet*** Physionet repository, the title of the Research Resource for Complex Physiologic Signals, was established in 1999 and is supported by the National Institutes of Health (NIH) (Goldberger et al. 2000). It is a widely used repository of biomedical data and contains datasets that can be used for PD diagnosis with gait data modality. This repository enables researchers to share and reuse clinical research resources and reduce barriers to data access.

### Clinical applicability

The clinical applicability of various diagnostic modalities for PD hinges on their practicality in real-world settings. Although neuroimaging techniques (DaTSCAN, SPECT) are useful in clinical diagnosis, they face limitations due to their high costs and the need for specialised equipment and trained personnel. These barriers make it less feasible to implement in low-resource Settings or routine screening. For EEG data, the subtleties associated with PD-related signal changes and the influences of various confounders, such as patient movement and electrical interference, complicate the interpretation of EEG results. Additionally, the absence of standardized protocols for EEG recording and analysis in the context of PD further complicates its widespread adoption in clinical practice. Conversely, voice, handwriting, and gait analyses offer a more accessible alternative, as they require minimal specialized equipment and can be performed remotely.

However, the clinical applicability of these modalities is contingent upon the standardization of data collection and the development of robust algorithms that can reliably interpret variations in patient data due to external factors such as background noise or emotional state. The adoption of voice and handwriting tools in clinical practice also depends on their integration into existing healthcare systems and workflows. For these tools to be widely accepted, they must demonstrate not only reliability and accuracy but also cost-effectiveness compared to more established diagnostic methods. PD poses a significant burden on both governments and patients’ families. As PD currently lacks a gold standard for diagnosis, ML tools are intended to serve as assistive tools, and their cost-effectiveness is crucial. Compared to MRI-based methods and EEG-based methods, voice, handwriting, and gait-based methods are more affordable and accessible. The integration with existing electronic health record (EHR) systems is also critical to ensure that AI models can be seamlessly embedded into current clinical workflows. To improve diagnostic precision and treatment planning, a database for PD patients with EHR should be established, which should contain a wide range of PD patient examination data, allowing for more personalised treatment. Lastly, for all diagnostic tools, including neuroimaging, voice, and handwriting analysis, there needs to be a clear regulatory pathway for their validation and approval. Establishing comprehensive guidelines that address privacy concerns, data security, and the ethical use of AI in clinical settings will be crucial for their broader adoption.

### Evaluation metrics

The evaluation metrics utilised in an ML classification task are Accuracy, Precision, Sensitivity(Recall), Specificity, Area Under Curve (AUC), Matthews Correlation Coefficient (MCC), and F1 score. For an actual positive class, if the result is a predicted positive class, it is a True Positive (TP); otherwise, it is a False Negative (FN). For an actual negative class, if the result is a predicted positive class, it is a False Positive (FP); otherwise, it is a True Negative (TN).

1 $$\begin{aligned} Accuracy= & \frac{TP + TN}{TP + TN + FP + FN} \end{aligned}$$

2 $$\begin{aligned} Precision= & \frac{TP}{TP + FP} \end{aligned}$$

3 $$\begin{aligned} Sensitivity= & \frac{TP}{TP + FN} \end{aligned}$$

4 $$\begin{aligned} Specificity= & \frac{TN}{TN + FP} \end{aligned}$$

5 $$\begin{aligned} \text {AUC }= & \text { the two-dimensional area under the Receiver Operating Characteristic (ROC) curve} \end{aligned}$$

6 $$\begin{aligned} MCC= & \frac{{Specificity}}{{Sensitivity}} \end{aligned}$$

7 $$\begin{aligned} F1\;score= & 2 \times \frac{{Precision} \times {Recall}}{{Precision} + {Recall}} \end{aligned}$$

**Table 1:** Summary of studies on PD classification using different modalities

Author	Year	Objective	Data modality	Dataset	Subjects	ML Algorithm	Validation	Evaluation metrics
*Neuroimaging*								
Prashanth et al. (2014)	2014	Classification (PD vs. HC)	Neuroimaging: DaTSCAN SPECT	PPMI	548 subjects: 369 PD + 179 HC	RBF-SVM	10-fold cross-validation	Accuracy: 96.14%, Sensitivity: 96.55%, Specificity: 95.03%
Salvatore et al. (2014)	2014	Classification (PD vs. HC)	Neuroimaging: MRI	Collected from participants	84 subjects: 56 PD + 28 HC	SVM	Leave-One-Out (LOO) validation	Accuracy: 92.2%, Sensitivity: 94.4%, Specificity: 91.3%
Rana et al. (2015)	2015	Classification (PD vs. HC)	Neuroimaging: MRI	Collected from participants	60 subjects: 30 PD + 30 HC	SVM	leave-one-out cross-validation (LOOCV)	Accuracy: 86.67%, Sensitivity: 90.00%, Specificity: 83.33%
Oliveira and Castelo–Branco (2015)	2016	Classification (PD vs. HC)	Neuroimaging: FP-CIT SPECT	PPMI	654 subjects: 445 PD + 209 HC	SVM	LOOCV	Accuracy: 97.68%, Sensitivity: 97.75%, Specificity: 98.09%
Zhang and Kagen (2017)	2017	Classification (PD vs. HC)	Neuroimaging: DaTSCAN SPECT	PPMI	Not specified	ANN	10-fold cross-validation	Accuracy: 93.8%, Sensitivity: 97.4%, Specificity: 82.2%
Peng et al. (2017)	2017	Classification (PD vs. HC)	Neuroimaging: MRI	PPMI	172 subjects: 69 PD + 103 HC	RBF-SVM	10-fold cross-validation	Accuracy: 85.8%, Sensitivity: 87.6%, Specificity: 87.8%
Sivaranjini and Sujatha (2020)	2020	Classification (PD vs. HC)	Neuroimaging: MRI	PPMI	182 subjects: 100 PD + 82 HC	AlexNet	Train and Test split (80–20%)	Accuracy: 88.90%, Sensitivity: 89.30%, Specificity: 88.40%
West et al. (2019)	2019	Classification (PD vs. HC)	Neuroimaging: MRI	PPMI	445 subjects: 299 PD + 146 HC	3D CNN	Not specified	Accuracy: 75%, Sensitivity: 76%, Specificity: 74%, Precision: 74%
Dai et al. (2019)	2019	Classification (PD vs. HC)	Neuroimaging: PET	PPMI, ANDI, HCP	Not specified	U-Net	10-fold cross-validation	Accuracy (U-Net): 84.17%, Accuracy (CNN): 76.19%
Zhang et al. (2019)	2019	Classification (Prodromal PD vs. Confirmed PD vs. HC)	Neuroimaging: MRI	PPMI	578 subjects: 49 Prodromal PD + 366 Confirmed PD + 163 HC	Deep neural network with Broad Views (DBV)	Train and Test split (80–20%)	Accuracy: 76.27%
Chakraborty et al. (2020)	2020	Classification (PD vs. HC)	Neuroimaging: MRI	PPMI	406 subjects: 203 PD + 203 HC	3D CNN	5-fold cross-validation	Accuracy: 95.29%, F1 score: 93.6%, Specificity: 94.3%, Precision: 92.7%, Recall: 94.3%, ROC-AUC: 98%
Kaur et al. (2021)	2021	Classification (PD vs. HC)	Neuroimaging: MRI	PPMI	Not specified	AlexNet	Train, Validation and Test split (60–20–20%)	Accuracy: 89.23%, Sensitivity: 90.27%, Specificity: 89.03%, ROC-AUC: 97.23%
Vyas et al. (2022)	2022	Classification (PD vs. HC)	Neuroimaging: MRI	PPMI	318 subjects: 236 PD + 82 HC	3D CNN	Train and Test split validation (70–30%)	3D CNN Accuracy: 88.9% 3D CNN AUC: 86.0%
Ya et al. (2022)	2022	Classification (PD vs. NC)	Neuroimaging: MRI	Collected from participants, PPMI	Collected from participants 116 subjects: 60 PD + 56 NC; PPMI 140 subjects: 69 PD + 71 NC	Regression models	5-fold cross-validation	Cerebellar model AUC: 64.6% Subcortical model AUC:63.2% Cortical model AUC:69.0% Combined model AUC:75.6%
Erdaş and Sümer (2022)	2022	Classification (PD vs. NC)	Neuroimaging: MRI	Combined from multiple datasets (Badea et al. 2017)	83 subjects: 47 PD + 36 NC	2D CNN	10-fold cross-validation	Accuracy: 90.36%, ROC-AUC: 90.51%, F1 score: 90.25%, Sensitivity: 90.52%, Precision: 90.08%
Huang et al. (2023)	2023	Classification (PD vs. HC)	Neuroimaging: MRI	PPMI	194 subjects: 97 PD + 97 HC	multi-task node cluster based graph structure learning framework (MNC-Net)	10-fold cross-validation	Accuracy: 95.5%, F1 score: 95.49%, Precision: 97.00%, Recall: 94.42%
Xu et al. (2023)	2023	Classification (PD vs. HC)	Neuroimaging: MRI	PPMI	117 subjects: 84 PD + 34 HC	DNN	5-fold cross-validation	Accuracy: 96.4%
Camacho et al. (2023)	2023	Classification (PD vs. HC)	Neuroimaging: MRI	PPMI	2041 subjects: 1024 PD + 1017 HC	CNN with Log-Jacobian model	Train, Validation, and Test split (85–5–10%)	Accuracy: 79.3%, Precision: 80.2%, Specificity: 81.3%, Sensitivity:77.7%
Priyadharshini et al. (2024)	2024	Classification (PD vs. HC)	Neuroimaging: 3D MRI	PPMI	500 subjects: 180 PD + 160 prodromal PD + 160 HC	Gradient Boosting (GB), with SHAP, LIME, SHAPASH for XAI	5-fold cross-validation	Accuracy: 96.8% Precision: 97% Recall: 94.2% Specificity: 96.6% F1 score: 94.6%
Talai et al. (2021)	2021	Classification (PD vs. PSP vs. HC)	Neuroimaging: T1, T2, DTI MRI	PPMI	103 subjects: 45 PD + 20 PSP-RS + 38 HC	SVM+MLP	LOOCV	Accuracy: 95.1%
Prasuhn et al. (2020)	2020	Classification (PD vs. HC)	Neuroimaging: Diffusion Tensor Imaging (DTI)	PPMI	232 subjects: 162 PD + 70 HC	SVM (bSVM)	10-fold cross-validation	Balanced Accuracy: 58.1% ROC-AUC: 52.0% Sensitivity: 56% Specificity:41%
Chen et al. (2023)	2023	Classification (PD-MCI vs. PD-NC)	Neuroimaging: DTI (FA, MD, AD, RD, LDH)	Collected from participants	117 subjects: 52 PD-NC + 68 PD-MCI	XGBoost	10-fold cross-validation	Accuracy: 91.67%, Sensitivity: 92.86%, Specificity: 90.00%, AUC: 94.00%
Tsai et al. (2023)	2023	Classification (PD vs. PSP vs. MSA vs. HC)	Neuroimaging: DTI (whole-brain features)	Collected from participants	625 subjects: 286 PD + 69 PSP + 51 MSA + 219 HC	SVM, Discriminant Function Analysis	5-fold cross-validation	Accuracy: 83.0%, Sensitivity: 84.8%, Specificity: 78.3%, F1 Score: 86.7%
Zhao et al. (2022)	2022	Classification (PD vs. HC)	Neuroimaging: DTI (Fractional Anisotropy, MD)	Collected from participants	532 subjects: 305 PD + 227 HC	3D CNN	10-fold cross-validation, independent test set	AUC: 94.1%
*Voice*								
Sakar and Kursun (2010)	2010	Classification (PD vs. HC)	Voice dataset	Oxford Parkinson’s Disease dataset	31 subjects: 23 PD + 8 HC	SVM	LOOCV	Accuracy: 81.53%, (LOO validation) Accuracy: 92.75% ( bootstrap resampling validation)
Bhattacharya and Bhatia (2010)	2010	Classification (PD vs. HC)	Voice dataset	Oxford Parkinson’s Disease dataset	31 subjects: 23 PD + 8 HC	Linear-SVM	Cross-validation	Accuracy: 65.22%
Guo et al. (2010)	2010	Classification (PD vs. HC)	Voice dataset	Oxford Parkinson’s Disease dataset	31 subjects: 23 PD + 8 HC	Minimum distance classifier (MDC)	10-fold cross-validation	Accuracy: 93.12%
Åström and Koker (2011)	2011	Classification (PD vs. HC)	Voice dataset	Oxford Parkinson’s Disease dataset	31 subjects: 23 PD + 8 HC	Parallel network system (9 FNN)	train and test split (60–40%)	Accuracy: 91.2% ± 1.6%
Ramani and Sivagami (2011)	2011	Classification (PD vs. HC)	Voice dataset	Oxford Parkinson’s Disease dataset	31 subjects: 23 PD + 8 HC	Fisher Filter + RF	Not specified	Accuracy: 100%
Yadav et al. (2012)	2012	Classification (PD vs. HC)	Voice dataset	Oxford Parkinson’s Disease dataset	31 subjects: 23 PD + 8 HC	SVM	10-fold cross-validation	Accuracy: 76%, Sensitivity: 97%, Specificity: 13%
Tsanas et al. (2012)	2012	Classification (PD vs. HC)	Voice dataset	NCVS	43 subjects: 33 PD + 10 HC	RELIEF + SVM	10-fold cross-validation	Accuracy: 98.6%
Mandal and Sairam (2014)	2014	Classification (PD vs. HC)	Voice dataset	Oxford Parkinson’s Disease dataset	31 subjects: 23 PD + 8 HC	LR	10-fold cross-validation	Accuracy: 100%, Sensitivity: 98.3%, Specificity: 99.6%
Hazan et al. (2012)	2012	Classification (PD vs. HC)	Voice dataset	Collected from participants	American Dataset: 52 subjects: 38 PD + 14 HC German Dataset: 98 subjects: 68 PD + 30 HC	SVM	Cross-validation	American Accuracy: 96%, German Accuracy: 85%
Gharehchopogh and Mohammadi (2013)	2013	Classification (PD vs. HC)	Voice dataset	Oxford Parkinson’s Disease dataset	31 subjects: 23 PD + 8 HC	MLP	train and test split (70–30%)	Accuracy: 93.22%
Rustempasic and Can (2013)	2013	Classification (PD vs. HC)	Voice dataset	Oxford Parkinson’s Disease dataset	31 subjects: 23 PD + 8 HC	MLP	Not specified	Accuracy: 81.33%
Sharma and Giri (2014)	2014	Classification (PD vs. HC)	Voice dataset	Oxford Parkinson’s Disease dataset	31 subjects: 23 PD + 8 HC	RBF-SVM	Train and Test split (80–20%)	Accuracy: 85.29%, Sensitivity: 100%, Specificity: 37.5%
Olanrewaju et al. (2014)	2014	Classification (PD vs. HC)	Voice dataset	Oxford Parkinson’s Disease dataset	31 subjects: 23 PD + 8 HC	MLFFN + K-Means	Train and Test split (50–50%)	Accuracy: 80%, Sensitivity: 63.6%, Specificity: 83.3%
Peker et al. (2015)	2015	Classification (PD vs. HC)	Voice dataset	Oxford Parkinson’s Disease dataset	31 subjects: 23 PD + 8 HC	CVANN	10-fold cross-validation	Accuracy: 98.12%, Sensitivity: 99.24%, Specificity: 98.96%
Gök (2015)	2015	Classification (PD vs. HC)	Voice dataset	Oxford Parkinson’s Disease dataset	31 subjects: 23 PD + 8 HC	Linear SVM + KNN	10-fold cross-validation	Accuracy: 98.46%
Chen et al. (2016)	2016	Classification (PD vs. HC)	Voice dataset	Oxford Parkinson’s Disease dataset	31 subjects: 23 PD + 8 HC	mRMR – KELM	10-fold cross-validation	Accuracy: 95.97%
Avci and Dogantekin (2016)	2016	Classification (PD vs. HC)	Voice dataset	Oxford Parkinson’s Disease dataset	31 subjects: 23 PD + 8 HC	GA-WK-ELM	3-fold cross-validation	Highest Accuracy: 96.81%
Dinesh and He (2017)	2017	Classification (PD vs. HC)	Voice dataset	Oxford Parkinson’s Disease dataset	31 subjects: 23 PD + 8 HC	Boosted Decision Tree	10-fold cross-validation	Highest Accuracy: 95%
Caliskan et al. (2017)	2017	Classification (PD vs. HC)	Voice dataset	Oxford Parkinson’s Disease dataset	31 subjects: 23 PD + 8 HC	DNN	10-fold cross-validation	Accuracy: 86.095%, Sensitivity: 58.27%, Specificity: 95.387%
Parisi et al. (2018)	2018	Classification (PD vs. HC)	Voice dataset	UCI Machine Learning repository	40 subjects: 20 PD + 20 HC	MLP-LSVM	20-fold cross-validation	Accuracy: 100%, Sensitivity: 100%, Specificity: 100%
Wroge et al. (2018)	2018	Classification (PD vs. HC)	Voice dataset	mPower dataset	N/A	SVM	10-fold cross-validation	Accuracy: 85%, Precision: 84%, Recall: 71%
Lahmiri et al. (2018)	2018	Classification (PD vs. HC)	Voice dataset	Private dataset	195 subjects: 147 PD + 48 HC	SVM	10-fold cross-validation	Accuracy: 92%, Sensitivity: 95%, Specificity: 91%
Haq et al. (2018)	2018	Classification (PD vs. HC)	Voice dataset	Oxford Parkinson’s Disease dataset	31 subjects: 23 PD + 8 HC	DNN	Train and Test split (70–30%)	Accuracy: 98%, Sensitivity: 95%, Specificity: 99%
Ali et al. (2019)	2019	Classification (PD vs. HC)	Voice dataset	UCI Machine Learning repository	40 subjects: 20 PD + 20 HC	LDA-NN-GA	leave-one-subject-out (LOSO) validation	Training Accuracy: 80%, Testing Accuracy: 82.14%
Mostafa et al. (2019)	2019	Classification (PD vs. HC)	Voice dataset	Oxford Parkinson’s Disease dataset	31 subjects: 23 PD + 8 HC	RF	10-fold cross-validation	Accuracy: 99.49%, Precision: 95.5%, Recall: 95.5%
Lahmiri and Shmuel (2019)	2019	Classification (PD vs. HC)	Voice dataset	Private dataset	43 subjects: 33 PD + 10 HC	Wilcoxon statistic + SVM	10-fold cross-validation	Accuracy: 92.21%, Sensitivity: 99.63%, Specificity: 82.79%
Haq et al. (2019)	2019	Classification (PD vs. HC)	Voice dataset	Oxford Parkinson’s Disease dataset	31 subjects: 23 PD + 8 HC	L1-norm SVM feature selection + SVM	10-fold cross-validation	Accuracy: 99%, Sensitivity: 100%, Specificity: 99%
Senturk (2020)	2020	Classification (PD vs. HC)	Voice dataset	Oxford Parkinson’s Disease dataset	31 subjects: 23 PD + 8 HC	SVM	Not specified	Accuracy: 93.84%
Karan et al. (2020)	2020	Classification (PD vs. HC)	Voice dataset	UCI Machine Learning repository + PC-GITA	UCI: 45 subjects: 25 PD + 20 HC PC-GITA: 45 subjects: 25 PD + 20 HC	SVM	10-fold cross-validation	UCI accuracy: 100%, PC-GITA accuracy: 96%
Soumaya et al. (2021)	2021	Classification (PD vs. HC)	Voice dataset	Collected from participants	34 subjects: 20 PD + 14 HC	GA + SVM	10-fold cross-validation	Best accuracy: 91.18%
Karaman et al. (2021)	2021	Classification (PD vs. HC)	Voice dataset	mPower dataset	N/A subjects	DenseNet-161	Not specified	Accuracy: 89.75% Specificity: 91.50% Sensitivity: 88.40%
Quan et al. (2021)	2021	Classification (PD vs. HC)	Voice dataset	Collected from participants	45 subjects: 30 PD + 15 HC	Bidirectional LSTM+CNN	10-fold cross-validation	Accuracy:75.56% F-score: 80.70% Specificity: 76.67% Sensitivity: 85.19% MCC: 0.4811
Zahid et al. (2020)	2020	Classification (PD vs. HC)	Voice dataset	pc-Gita dataset	100 subjects: 50 PD + 50 HC	AlexNet	5-fold cross-validation	Accuracy (RF): 99%, Accuracy (MLP): 99.7%
Rizvi et al. (2020)	2020	Classification (PD vs. HC)	Voice dataset	PSD dataset	40 subjects: 20 PD + 20 HC	LSTM + DNN	Not specified	Accuracy: 99.03%, Sensitivity: 99%, Specificity: 99%, Precision: 99%
Abayomi-Alli et al. (2020)	2020	Classification (PD vs. HC)	Voice dataset	Oxford Parkinson’s Disease dataset	31 subjects: 23 PD + 8 HC	Bidirectional LSTM	5-fold cross-validation	Accuracy: 82.86%
Gunduz (2019)	2019	Classification (PD vs. HC)	Voice dataset	UCI Machine Learning repository	252 subjects: 188 PD + 64 HC	2D CNN	Leave-one-person-out cross-validation	Accuracy (Triple feature sets): 83.3% F-score (Triple feature sets): 89.4% MCC (Triple feature sets): 0.521
Nagasubramanian and Sankayya (2021)	2021	Classification (PD vs. HC)	Voice dataset	Parkinson telemonitoring dataset + multi-variate sound record dataset	102 subjects: 82 PD + 20 HC	DWVDA	Not specified	Accuracy (ADNN): 98.96% Specificity (ADNN): 98.82% Recall (ADNN): 98.89% Precision (ADNN): 98.90% MAE(ADNN): 1.04
Fang et al. (2020)	2020	Classification (PD vs. HC)	Voice dataset	Collected from participants	68 subjects: 34 PD + 34 HC	CNN + LSTM	LOSO validation	ACC (Talking): 94.0% ACC (DDK): 83.5% ACC (Reading): 91.1%
Ali et al. (2023)	2023	Classification (PD vs. HC)	Voice dataset	Combined from two public datasets	228 subjects: 108 PD + 120 HC	Ensemble learning-based framework	LOSO	Accuracy: 100%
Hireš et al. (2022)	2022	Classification (PD vs. HC)	Voice dataset	PC-GITA dataset	100 subjects: 50 PD + 50 HC	2D CNN	10-fold cross-validation	Accuracy: 99%, AUC: 99.6%, Sensitivity: 86.2%, Specificity: 93.3%
Rana et al. (2022)	2022	Classification (PD vs. HC)	Voice dataset	Oxford Parkinson’s Disease dataset	195 subjects: 147 PD + 48 HC	ANN	train and test split (80–20%)	Accuracy (SVM): 87.2%, Accuracy (NB): 74.1%, Accuracy (ANN): 96.7%, Accuracy (KNN): 87.2%
Madruga et al. (2023)	2023	Classification (PD vs. HC)	Voice dataset	Collected from participants	60 subjects: 30 PD + 30 HC	Passive aggressive classifier	Cross-validation	Accuracy (position 1): 70.1%, Accuracy (position 2): 71.8%, Accuracy (position 3): 72.9%, Accuracy (position 4): 73.1%
Govindu and Palwe (2023)	2023	Classification (PD vs. HC)	Voice dataset	Oxford Parkinson’s Disease dataset	31 subjects: 23 PD + 8 HC	RF	train and test split (75–25%)	Accuracy: 91.8%, Precision: 95.0%, Recall: 86.0%
Celik and Başaran (2023)	2023	Classification (PD vs. HC)	Voice dataset	PD Dataset and PDO Dataset	PD Dataset: 252 subjects (188 PD + 64 HC) PDO Dataset: 31 subjects (23 PD+ 8 HC)	SkipCon Net + RF	Not specified	Accuracy: 99.1%, Precision: 99.0%, Recall: 99.0%, Specificity: 98%, Specificity:98.77%
Khaskhoussy and Ayed (2023)	2023	Classification (PD vs. HC)	Voice dataset	UCI Machine Learning repository	40 subjects: 20 PD + 20 HC	Polynomial kernel SVM	5-fold cross-validation	Accuracy: 97.6%, Precision: 94%, Sensitivity: 96%, Specificity: 93%, F-Score: 94%
Dheer et al. (2023)	2023	Classification (PD vs. HC)	Voice dataset	Oxford Parkinson’s Disease dataset	31 subjects: 23 PD + 8 HC	KNN	train and test split (75–25%)	Accuracy: 95.9%
Akila and Nayahi (2024)	2024	Classification (PD vs. HC)	Voice dataset	UCI Machine Learning repository	252 subjects: 188 PD + 64 HC	MASS-PCNN (Multi-agent Salp Swarm Algorithm)	5-fold cross-validation	Accuracy: 95.1%, Precision: 97.8%, Recall: 94.7%, F1 score: 99.1%
*Handwriting*								
Drotár et al. (2014)	2014	Classification (PD vs. HC)	Handwriting dataset	Collected from participants	75 subjects: 37 PD + 38 HC	SVM	10-fold cross-validation	Accuracy: 95.29%, F1 score: 93.6%, Specificity: 94.3%, Precision: 92.7%, Recall: 94.3%, ROC-AUC: 98%
Drotár et al. (2015)	2015	Classification (PD vs. HC)	Handwriting dataset	Collected from participants	75 subjects: 37 PD + 38 HC	RBF-SVM	10-fold cross-validation	Accuracy: 88.1%
Pereira et al. (2015)	2015	Classification (PD vs. HC)	Handwriting dataset	Collected from participants	55 subjects: 37 PD + 18 HC	NB	10-fold cross-validation	Accuracy: 88.13%, Sensitivity: 89.74%, Specificity: 91.89%
Ribeiro et al. (2019)	2019	Classification (PD vs. HC)	Handwriting dataset	HandPD dataset	35 subjects: 14 PD + 21 HC	Gated Recurrent Units + Attention	train and test split (75–25%)	Accuracy: 78.9%
Razzak et al. (2020)	2020	Classification (PD vs. HC)	Handwriting dataset	PaHaW, NewHan dataset, Parkinson’s Drawing Dataset	233 subjects: 142 PD + 91 HC	2D CNN (AlexNet, GoogleNet, VGGNet, ResNet)	10-fold cross-validation	Accuracy: 89.48%
Kamran et al. (2021)	2021	Classification (PD vs. HC)	Handwriting dataset	HandPD, NewHandPD, PaHaw, Parkinson’s Drawing Dataset	233 subjects: 142 PD + 91 HC Parkinson’s Drawing Dataset: NA	2D CNN	5-fold cross-validation	Accuracy: 98.04%
Gil-Martín et al. (2019)	2019	Classification (PD vs. HC)	Handwriting dataset	Spiral Drawing dataset	77 subjects: 62 PD + 15 HC	2D CNN	subject-wise 5-fold cross-validation	Accuracy: 96.5%, F1 score: 97.7%, AUC: 99.2%
Diaz et al. (2021)	2021	Classification (PD vs. HC)	Handwriting dataset	PaHaW, NewHan dataset	75 subjects: 37 PD + 38 HC	BiGRUs + CNN	10-fold cross-validation	Accuracy: 94.44%, AUC: 98.25%, Specificity: 98.0%, Sensitivity: 90.0%
Taleb et al. (2019)	2019	Classification (PD vs. HC)	Handwriting dataset	PDMulti MC dataset	42 subjects: 21 PD + 21 HC	CNN + CNN-BLSTM	3-fold cross-validation	Accuracy: 83.33%, Sensitivity: 71.43%, Specificity: 95.24%
Varalakshmi et al. (2022)	2022	Classification (PD vs. HC)	Handwriting dataset	Kaggle spiral data	51 subjects: 50 healthy + 1 PD	A hybrid of RESNET-50 and SVM	train and test split (70–30%)	Accuracy: 98.45%, Sensitivity: 99%, Specificity: 99%
Li et al. (2022)	2022	Classification (PD vs. HC)	Handwriting dataset	Collected from participants	86 subjects: 43 PD + 43 HC	CNN (CC-Net)	Cross-validation	Accuracy: 89.3%, Precision: 99.2%, Recall: 93.1%, F1 Score: 92.5%, Matthews correlation coefficient (MCC): 73.3%
Zhao and Li (2023)	2023	Classification (PD vs. HC)	Handwriting dataset	NewHan dPD	66 subjects: 31 PD + 35 HC	CNN and bidirectional gated recurrent unit (BiGRU)	train and test split (80–20%)	Accuracy (meander): 92.91%, Accuracy (circle): 85.71%, Accuracy (spiral): 90.55%
Abdullah et al. (2023)	2023	Classification (PD vs. HC)	Handwriting dataset	NewHan dPD	66 subjects: 31 PD + 35 HC	ResNet5+ VGG19+Inception V3+kNN	train and test split (80–20%)	Accuracy: 95.29%, AUC: 90%, Recall: 86%, Precision: 99%
Wang et al. (2024)	2024	Classification (PD vs. HC)	Handwriting dataset	DraWritePD, PaHaW datasets	75 subjects: 37 PD + 38 HC	LSTM-CNN	5-fold cross-validation	Accuracy: 96.2%, Sensitivity: 94.5%, Specificity: 97.3%, PaHaW Accuracy: 90.7%
*Gait*								
Tahir and Manap (2012)	2012	Classification (PD vs. HC)	Gait dataset	Collected from participants	32 subjects: 12 PD + 20 HC	SVM	10-fold cross-validation	Accuracy: 100%, Sensitivity: 100%, Specificity: 100%
Wahid et al. (2015)	2015	Classification (PD vs. HC)	Gait dataset	Collected from participants	49 subjects: 23 PD + 26 HC	RF	10-fold cross-validation	Accuracy: 92.6%
Shetty and Rao (2016)	2016	Classification (PD vs. HD vs. ALS)	Gait dataset	Physionet dataset	48 subjects: 15 PD + 20 HD + 13 ALS	SVM	train and test split (50–50%)	Accuracy: 83.33%, Sensitivity: 85.71%, Specificity: 75%
Abdulhay et al. (2018)	2018	Classification (PD vs. HC)	Gait dataset	Physionet dataset	166 subjects: 93 PD + 73 HC	Medium Gaussian SVM	Not specified	Accuracy: 94.8%
Rehman et al. (2019)	2019	Classification (PD vs. HC)	Gait dataset	Collected from participants	303 subjects: 119 PD + 184 HC	RF	10-fold cross-validation	Accuracy: 97%, Sensitivity: 100%, Specificity: 94%
Balaji et al. (2021)	2021	Classification (PD vs. HC)	Gait dataset	Physionet dataset	166 subjects: 93 PD + 73 HC	LSTM	train and test split (80–20%)	Accuracy: 98.6%
Xia et al. (2019)	2019	Classification (PD vs. HC)	Gait dataset	Physionet dataset	166 subjects: 93 PD + 73 HC	CNN, Attention-enhanced LSTM	5-fold cross-validation	Accuracy: 99.07% Sensitivity: 99.10% Specificity: 99.01%
El Maachi et al. (2020)	2020	Classification (PD vs. HC)	Gait dataset	Physionet dataset	166 subjects: 93 PD + 73 HC	DNN	10-fold cross-validation	Accuracy: 98.7%
Aversano et al. (2020)	2020	Classification (PD vs. HC)	Gait dataset	Physionet dataset	166 subjects: 93 PD + 73 HC	DNN	10-fold cross-validation	Accuracy: 99.37%
Liu et al. (2021)	2021	Classification (PD vs. HC)	Gait dataset	Physionet dataset	166 subjects: 93 PD + 73 HC	CNN with Bi-LSTM	Train and Test split (70–30%)	Accuracy: 99.22%, Sensitivity: 100%, Specificity: 98.04%
Nguyen et al. (2022)	2022	Classification (PD vs. HC)	Gait dataset	Physionet	166 subjects: 93 PD + 73 HC	Transformer	10-fold cross-validation	Accuracy: 95.2%, Sensitivity: 98.1%, Specificity: 86.8%
Trabassi et al. (2022)	2022	Classification (PD vs. HC)	Gait dataset	Collected from participants	161 subjects: 81 PD + 80 HC	SVM	10-fold cross-validation, Train and Test split (80–20%)	Accuracy: 81% AUC: 80% F1 score:80% Precision: 80% Recall: 80%
Li and Li (2022)	2022	Classification (PD vs. HC)	Gait dataset	Two public datasets	306 subjects: 214 PD + 92 HC	SVM	Train and Test split (80–20%)	Accuracy: 68% False positive rate: 98% Precision: 69% Recall: 98%
Aşuroğlu and Oğul (2022)	2022	Classification (PD vs. HC), Regression (UPDRS value)	Gait dataset	Physionet	166 subjects: 93 PD + 73 HC	CNN + RF	10-fold cross-validation	Accuracy: 99.5% Sensitivity: 98.7% Specificity: 99.1% Correlation Coefficient: 0.897 Mean Absolute Error: 3.009 Root Mean Square Error: 4.556
Ma et al. (2023)	2023	Classification (PD vs. HC)	Gait dataset	Physionet	166 subjects: 93 PD + 73 HC	CNN+XGBoost	Train and Test split	Accuracy: 98.4%
Vinora et al. (2023)	2023	Classification (PD vs. HC)	Gait dataset	UCI Machine Learning repository	85 subjects: 70 PD + 15 HC	SVM	Not specified	Recall: 100%, Precision: 50%, F1 score: 67%
Sharma et al. (2023)	2023	Classification (PD vs. HC)	Gait dataset	Physionet dataset	166 subjects: 93 PD + 73 HC	CNN+SVM	10-fold cross-validation	Accuracy: 95.2%
*EEG*								
Lee et al. (2019)	2019	Classification (PD vs. HC)	EEG	Collected from participants	406 subjects: 203 PD + 203 HC	3D CNN	Train and Test split (80–20%)	Accuracy: 95.29% F1 score: 93.6% Specificity: 94.3% Precision: 92.7% Recall: 94.3% ROC-AUC: 98%
Oh et al. (2020)	2020	Classification (PD vs. HC)	EEG	Collected from participants	41 subjects: 20 PD + 21 HC	CNN + LSTM	10-fold cross-validation	Accuracy: 96.9%, Recall: 93.4%, Precision: 100%
Anjum et al. (2020)	2020	Classification (PD vs. HC)	EEG	Collected from participants	Participants from New Mexico 54 subjects: 27 PD + 27 HC Participants from Iowa 28 subjects: 14 PD + 14 HC	Linear predictive coding	10-fold cross-validation	Accuracy: 85.3%, AUC: 93.3%, Sensitivity: 87.9%, Specificity: 82.7%
Shaban (2021)	2021	Classification (PD vs. HC)	EEG	UC San Diego Public Dataset	31 subjects: 16 PD + 15 HC	ANN	Train and Test split (80–20%)	Accuracy: 98%, Sensitivity: 97%, Specificity: 100%
Loh et al. (2021)	2021	Classification (PD vs. HC)	EEG	UC San Diego Public Dataset	31 subjects: 16 PD + 15 HC	2D-CNN	10-fold cross-validation	Accuracy: 99.46%
Motin et al. (2022)	2022	Classification (PD vs. HC)	EEG	UC San Diego Public Dataset	31 subjects: 16 PD + 15 HC	Polynomial SVM	Train and Test split	Accuracy: 87.1%, Sensitivity: 93.3%, Specificity: 81.25%
Chawla et al. (2023)	2023	Classification (PD vs. HC)	EEG	Combined from two public datasets	Dataset-1 40 subjects: 20 PD + 20 HC Dataset-2 31 subjects: 16 PD + 15 HC	flexible analytic wavelet transform (FAWT) + KNN	10-fold cross-validation	Dataset-1 Accuracy: 99% AUC: 99.1% Sensitivity: 99.12% Specificity: 99.45% Dataset-2 Accuracy: 95.85% AUC: 95.9% Sensitivity: 96.14% Specificity: 95.88%
Coelho et al. (2023)	2023	Classification (PD vs. HC)	EEG	Public PRED+C repository	50 subjects: 25 PD + 25 HC	SVM	5-fold cross-validation	Accuracy: 89.56%
Nour et al. (2023)	2023	Classification (PD vs. HC)	EEG	UC San Diego Public Dataset	31 subjects: 16 PD + 15 HC	Dynamic Classifier Selection (DCS) in Modified Local Accuracy (MLA)	5-fold cross-validation	Accuracy: 99.3%, Precision: 99.31%, Recall: 99.31%
Zhao et al. (2024)	2024	Classification (PD vs. HC)	EEG	Collected from participants	100 subjects: 52 PD + 48 HC	GSP-GCNs (Graph Signal Processing-Graph Convolutional Networks)	5-fold cross-validation	Accuracy: 90.2%, AUC: 89.1%, Sensitivity: 84.0%, Specificity: 88.4%
*Other Data*								
Bhandari et al. (2023)	2023	Classification (PD vs. HC)	Gene dataset	Five open-source peripheral blood microarray gene expression datasets on PD from GEO	742 subjects: 406 PD + 336 HC	Logistic Regression	10-fold cross-validation	Accuracy: 77.7%, Precision: 77.6%, Recall: 77.82%
Wang et al. (2023)	2023	Classification (PD vs. HC)	Urine biomarkers	Collected from participants	215 subjects: 104 PD + 111 HC	XGBoost	Not specified	Accuracy: 96.5%, AUC: 99.2%
Junaid et al. (2023)	2023	Classification (PD vs. HC)	Patient visits	PPMI	215 subjects: 324 PD + 217 HC	Light gradient boosting machines (LGBM)	10-fold cross-validation	Accuracy: 90.73%, Precision: 83.27%, Recall: 89.53%
Igene et al. (2023)	2023	Classification (PD vs. HC)	Movement data	Collected from participants	34 subjects: 17 PD + 17 HC	SVM	10-fold cross-validation	Accuracy: 94.4%
Varghese et al. (2024)	2024	Classification (PD vs. HC)	Smartwatch data, Questionnaire data	PADS (PD Smartwatch) dataset	469 subjects: 276 PD + 114 DD + 79 HC	Classifier stacking (SVM, NN, CatBoost, Xception- Time)	Nested 5-fold cross-validation	Accuracy: 91.16%, Precision: 96.98%, Recall: 92.40%, F1 score: 94.62%

HC: health control, NC: normal control, UPDRS: Unified PD Rating Scale, CNN: convolutional neural network, RNN: recurrent neural network, MLP: multilayer perceptron, DT: decision tree, SVM: support vector machine, ANN: Artificial neural network, RF: random forest, LR: linear regression, NB: Naïve Bayes

## Risk of bias

The risk of bias is assessed using the Prediction Model Risk of Bias Assessment Tool (PROBAST) (Wolff et al. 2019). PROBAST is designed to evaluate the risk of bias in the diagnostic model study. In this review, the risk of bias in all included studies is assessed independently and then validated by the authors separately. The results of the risk of bias assessment are shown in Table 2. Most of the studies are at high risk of bias or unclear, and 28 studies are at low risk of bias (Ya et al. 2022; Huang et al. 2023; Xu et al. 2023; Camacho et al. 2023; Peker et al. 2015; Chen et al. 2016; Parisi et al. 2018; Ali et al. 2019; Haq et al. 2019; Li et al. 2022; Zhao and Li 2023; Abdullah et al. 2023; Balaji et al. 2021; Xia et al. 2019; Oh et al. 2020; Trabassi et al. 2022; Anjum et al. 2020; Chawla et al. 2023; Coelho et al. 2023; Khaskhoussy and Ayed 2023; Nour et al. 2023; Junaid et al. 2023; Zhao et al. 2024; Priyadharshini et al. 2024; Wang et al. 2024; Akila and Nayahi 2024; Hireš et al. 2022; Tsai et al. 2023).

**Fig. 11:**
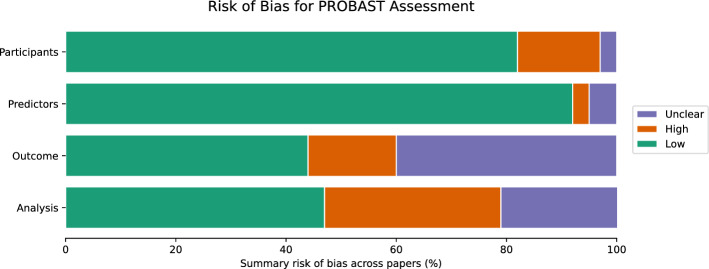
Risk of bias PROBAST assessment summary

We follow the standard PROBAST framework, which evaluates studies across four domains: participants, predictors, outcome, and analysis. We have found that many included studies used small datasets, which limit the generalizability of their findings. Moreover, several studies had methodological flaws, including data leakage, insufficient sample sizes, and unrealistic validation protocols. These issues contribute to a high risk of bias, particularly in the Participants and Analysis domains of the PROBAST framework. For example, in the study (Sivaranjini and Sujatha 2020), a high risk of bias was identified in the Analysis domain. This was due to the study only reporting the experimental results without providing a detailed analysis of the dataset or methodological details. Moreover, the study used MRI data and applied an image-level train-test split rather than subject-level cross-validation, which increased the likelihood of data leakage. As a result, the study was assessed as having a high risk of bias in the “Analysis” domain, and the overall risk of bias was deemed high. Fig. 11 shows the PROBAST evaluation results in a heatmap.

While certain data modalities are indeed associated with a higher risk of bias, they nonetheless demonstrate substantial potential for ML-based PD diagnostics. In particular, EEG and gait signals stand out due to their biological plausibility, accessibility, and practical advantages in clinical settings.

EEG offers high temporal resolution and captures neurophysiological activity directly linked to both motor dysfunction and cognitive impairment, two hallmark features of PD. Likewise, gait analysis reflects core motor symptoms such as bradykinesia, rigidity, and postural instability, making it a valuable modality for both diagnosis and monitoring of disease progression. Importantly, these modalities align well with clinicians’ existing understanding of PD pathophysiology and assessment practices, which may facilitate greater acceptance and integration into clinical workflows.

**Table 2:** Risk of bias assessment of the included studies according to the PROBAST checklist. “+” indicates a low risk of bias, “-” indicates a high risk of bias, and “?” means an unclear risk of bias

#	Study	Participants	Predictors	Outcome	Analysis	Risk of bias
1	West et al. (2019)	+	+	−	?	−
2	Dai et al. (2019)	?	+	−	+	−
3	Zhang et al. (2019)	+	+	−	?	−
4	Chakraborty et al. (2020)	+	+	?	+	?
5	Kaur et al. (2021)	+	+	?	+	?
6	Vyas et al. (2022)	+	+	?	+	?
7	Quan et al. (2021)	+	+	?	?	?
8	Zahid et al. (2020)	+	+	?	+	?
9	Rizvi et al. (2020)	+	+	−	−	−
10	Abayomi-Alli et al. (2020)	+	+	?	−	−
11	Gunduz (2019)	+	+	?	+	?
12	Nagasubramanian and Sankayya (2021)	+	+	?	−	−
13	Fang et al. (2020)	+	+	?	?	?
14	Ribeiro et al. (2019)	+	+	?	?	?
15	Razzak et al. (2020)	+	+	?	?	?
16	Kamran et al. (2021)	+	+	?	?	?
17	Gil-Martín et al. (2019)	+	+	?	?	?
18	Diaz et al. (2021)	+	+	?	?	?
19	Taleb et al. (2019)	+	+	?	?	?
20	Xia et al. (2019)	+	+	+	+	+
21	El Maachi et al. (2020)	+	+	?	?	?
22	Aversano et al. (2020)	+	+	?	−	−
23	Liu et al. (2021)	+	+	?	+	?
24	Lee et al. (2019)	+	+	?	?	?
25	Oh et al. (2020)	+	+	+	+	+
26	Shaban (2021)	+	+	+	?	?
27	Loh et al. (2021)	+	+	−	?	−
28	Prashanth et al. (2014)	+	+	?	?	?
29	Salvatore et al. (2014)	+	+	?	?	?
30	Rana et al. (2015)	+	+	?	?	?
31	Oliveira and Castelo-Branco (2015)	+	+	?	?	?
32	Zhang and Kagen (2017)	+	+	?	?	?
33	Peng et al. (2017)	+	+	?	+	?
34	Sivaranjini and Sujatha (2020)	+	+	+	−	−
35	Sakar and Kursun (2010)	+	+	?	+	?
36	Bhattacharya and Bhatia (2010)	+	?	−	–	–
37	Guo et al. (2010)	+	+	+	?	?
38	Åström and Koker (2011)	+	+	?	+	?
39	Ramani and Sivagami (2011)	+	+	?	?	?
40	Yadav et al. (2012)	–	+	?	+	−
41	Tsanas et al. (2012)	+	+	?	+	?
42	Mandal and Sairam (2014)	+	+	?	+	?
43	Hazan et al. (2012)	−	+	?	−	−
44	Gharehchopogh and Mohammadi (2013)	+	+	?	?	?
45	Rustempasic and Can (2013)	+	+	?	−	−
46	Sharma and Giri (2014)	+	+	+	−	−
47	Olanrewaju et al. (2014)	+	+	?	?	?
48	Peker et al. (2015)	+	+	+	+	+
49	Gök (2015)	+	+	+	?	?
50	Chen et al. (2016)	+	+	+	+	+
51	Avci and Dogantekin (2016)	?	+	?	−	−
52	Dinesh and He (2017)	+	?	?	−	−
53	Caliskan et al. (2017)	+	+	+	−	−
54	Parisi et al. (2018)	+	+	+	+	+
55	Wroge et al. (2018)	?	+	?	?	?
56	Lahmiri et al. (2018)	+	+	?	+	?
57	Haq et al. (2018)	+	+	+	−	−
58	Ali et al. (2019)	+	+	+	+	+
59	Mostafa et al. (2019)	+	+	?	?	?
60	Lahmiri and Shmuel (2019)	+	+	?	+	?
61	Haq et al. (2019)	+	+	+	+	+
62	Senturk (2020)	+	+	?	−	−
63	Karan et al. (2020)	+	+	?	+	?
64	Soumaya et al. (2021)	−	+	+	?	−
65	Karaman et al. (2021)	+	+	−	+	−
66	Drotár et al. (2014)	+	+	−	+	−
67	Drotár et al. (2015)	+	+	−	−	−
68	Pereira et al. (2015)	+	?	−	−	−
69	Tahir and Manap (2012)	+	+	−	+	−
70	Wahid et al. (2015)	+	+	?	+	?
71	Shetty and Rao (2016)	+	+	−	?	−
72	Abdulhay et al. (2018)	+	+	−	?	−
73	Rehman et al. (2019)	+	+	?	+	?
74	Balaji et al. (2021)	+	+	+	+	+
75	Ya et al. (2022)	+	+	+	+	+
76	Erdaş and Sümer (2022)	−	?	+	+	−
77	Huang et al. (2023)	+	+	+	+	+
78	Ali et al. (2023)	−	−	+	+	−
79	Hireš et al. (2022)	+	+	+	+	+
80	Rana et al. (2022)	−	+	+	−	−
81	Madruga et al. (2023)	−	?	+	−	−
82	Varalakshmi et al. (2022)	−	+	+	−	−
83	Li et al. (2022)	+	+	+	+	+
84	Zhao and Li (2023)	+	+	+	+	+
85	Abdullah et al. (2023)	+	+	+	+	+
86	Nguyen et al. (2022)	+	+	+	−	−
87	Trabassi et al. (2022)	+	+	+	+	+
88	Li and Li (2022)	−	+	+	−	−
89	Aşuroğlu and Oğul (2022)	+	+	+	−	−
90	Ma et al. (2023)	−	−	+	−	−
91	Anjum et al. (2020)	+	+	+	+	+
92	Motin et al. (2022)	−	−	+	−	−
93	Chawla et al. (2023)	+	+	+	+	+
94	Coelho et al. (2023)	+	+	+	+	+
95	Xu et al. (2023)	+	+	+	+	+
96	Camacho et al. (2023)	+	+	+	+	+
97	Govindu and Palwe (2023)	+	+	−	−	−
98	Celik and Başaran (2023)	+	+	−	−	−
99	Khaskhoussy and Ayed (2023)	+	+	+	+	+
100	Dheer et al. (2023)	−	−	−	−	−
101	Vinora et al. (2023)	−	+	−	−	−
102	Sharma et al. (2023)	−	+	+	+	−
103	Nour et al. (2023)	+	+	+	+	+
104	Bhandari et al. (2023)	−	+	+	−	−
105	Wang et al. (2023)	+	+	−	−	−
106	Junaid et al. (2023)	+	+	+	+	+
107	Igene et al. (2023)	−	+	−	−	−
108	Varghese et al. (2024)	+	+	+	?	?
109	Zhao et al. (2024)	+	+	+	+	+
110	Priyadharshini et al. (2024)	+	+	+	+	+
111	Wang et al. (2024)	+	+	+	+	+
112	Akila and Nayahi (2024)	+	+	+	+	+
113	Talai et al. (2021)	+	+	?	?	?
114	Prasuhn et al. (2020)	+	+	−	?	−
115	Chen et al. (2023)	?	+	?	+	?
116	Tsai et al. (2023)	+	+	+	+	+
117	Zhao et al. (2022)	+	+	?	−	−

## Case studies

In this paper, we have done 5 case studies (1 for MRI, 1 for gait, 1 for voice, 1 for EEG, 1 for handwriting). We repeat the experiment to reproduce the result provided in these papers (Table 3). In our reproduction experiments, we adopted a unified evaluation framework using the following metrics: Accuracy, Specificity, Sensitivity, Precision, Recall, F1 score, AUC (Area Under the ROC Curve), RGA (Ranked Graduation Accuracy) (Giudici and Raffinetti 2025), Lorenz Zonoid (Calzarossa et al. 2025), RGR (Rank Graduation Robustness) (Babaei et al. 2025).

### Case study 1: voice

Parkinson Speech Dataset: The dataset was collected by Sarkar et al. at the Department of Neurology in Cerrahpasa, Faculty of Medicine, Istanbul University. The dataset can be divided into two parts: training and testing. The training dataset includes data from 20 PD patients and 20 healthy subjects. The age of PD patients is between 43 and 77 years, while healthy subjects are aged between 45 and 83 years. From each subject, 26 samples were recorded. For the testing part, it contains data from 28 subjects (all PD patients) aged between 39 and 79 years. For each subject, 6 samples were recorded.

Data Preprocessing: LDA is used to reduce the data dimension. It transforms the original feature vectors into the reduced vector space where the class separability is maximised.

Result: LOSO validation is used to evaluate the model’s performance. The source code is provided in https://github.com/LiaqatAli007/Automated−Detection-of-Parkinson-s-Disease-Based-on-Multiple-Types-of-Sustained-Phonations-using-Lin. There is only one class, “PD”, in the test set. The paper reported that the model can achieve a 100% accuracy, and the result of the reproduction experiment matches the result reported by the paper.

### Case study 2: gait

Physionet Dataset: The dataset was collected from three research (Yogev et al. 2005; Frenkel-Toledo et al. 2005; Goldberger et al. 2000; Hausdorff et al. 2007). 93 Parkinson’s patients (mean age: 66.3 years; 63% men) and 73 healthy controls (mean age: 66.3 years; 55% men) are included in the dataset. For each subject, there are 8 sensors on each foot with a 2-minute length measure of the vertical ground reaction force (in Newtons). The output of each sensor is digitised and recorded at 100 samples per second. Two extra signals reflect the sum of the 8 sensor outputs for each foot.

Data Preprocessing: Each 1D signal is divided into smaller segments with a length of 100 time steps and 50

Result: 10-fold cross-validation is used to evaluate the model’s performance. There are two groups: PD and HC. Each of them is divided into 10 folds at the subject level and combined to form a fold with 70% Parkinson and 30% control. The source code is provided at https://github.com/DucMinhDimitriNguyen/Transformers-for-1D-signals-in-Parkinson-s-disease-detection-from-gait. The result report by the paper is 98.1% in sensitivity, 86.8% in specificity, and 95.2% in accuracy. However, the result of the reproduction experiment cannot achieve the reported performance. It achieved a sensitivity of 94.03%, specificity of 68.24%, and accuracy of 87.12%.

### Case study 3: EEG

Dataset: The dataset was collected by the Aron lab at the University of California, San Diego, and subsequently further analyzed by the Swann lab at the University of Oregon. There are 16 PD patients (8 females; mean age: 62.6±8.3) and 15 HC (9 females; mean age: 63.5±9.6) included in the dataset. The data was captured using 40 electrodes with a sampling rate of 512Hz.

Data Preprocessing: Select the data in channels of $\textit{O}_{z}$, $\textit{P}_{8}$, and $\textit{FC}_{2}$.9s to 2 minutes and segmented into patches of 512 time samples.

Result: The dataset is divided into three parts: 64% for train, 16% for validation, and 20% for test. There is no source code provided, only the model structure. The paper reported that it can achieve an accuracy of 98.00%, sensitivity of 97.00%, and specificity of 100.00%. However, according to our reproduction, it only achieves 62.40% accuracy, 62.10% sensitivity, and 62.68% specificity. The reason may be that the author used the pre-trained model.

### Case study 4: handwriting

NewHandPD Dataset: The dataset was collected by the Botucatu Medical School, São Paulo State University. It contains 12 exams (4 of them related to spirals, 4 related to meanders, 2 circled movements, and left and right-handed diadochokinesis). There are 31 PD patients (10 females; mean age: 57.83±7.85) and 35 HC (17 females; mean age: 44.05±14.88) included in the dataset.

Data Preprocessing: The 5th and 90th percentiles were set as lower and upper bounds. Values outside these bounds were replaced by boundary values to mitigate outlier effects. Normalisation is applied to have a zero mean and unitary standard deviation.

Result: The dataset is divided into three parts: 60% for training, 15% for validation, and 25% for testing. The source code is provided in https://github.com/lzfelix/bag-of-samplings. The paper reported that it can achieve an accuracy of 89.48%±3.7%, precision of 84.8%±4.7%, recall of 95.5%±4.8%, and F1 score of 89.7%±3.5% in the Spiral dataset and an accuracy of 92.24%±2.65%, precision of 95.2%±2.5%, recall of 88.3%±4.9%, and F1 Score of 92.4%±3.1% in the Meander dataset. However, according to our reproduction, it only achieves 84.03%±2.67% accuracy, 86.79%±6.60% precision, 79.69%±7.30% recall and 82.61%±2.92% F1 score in the Spiral dataset and 79.40%±3.52% accuracy, 86.95%±3.57% precision, 67.19%±8.76% recall and 75.41%±5.38% F1 score in the Meander dataset.

### Case study 5: MRI

Dataset: The dataset was created by Badea et al. (2017), which combined the T1 MRI images from two datasets collected by Neurocon and Taowu. There are 83 subjects included in the dataset, with 43 from Neurocon (27 PD patients and 16 controls) and 40 from Taowu (20 PD patients and 20 controls).

Data Preprocessing: Median slices from the axial, coronal, and sagittal planes of 3D MR images were extracted and resized to 224x224 pixels. The three median slices are combined into a single three-channel image to maintain spatial integrity across different planes.

Result: 10-fold cross-validation is used to evaluate the model’s performance. The source is not provided, but we reproduce the experiment based on the provided model architecture. The paper reported that it can achieve an accuracy of 90.36%, precision of 90.08%, sensitivity of 90.52%, AUC of 90.51%, and F1 Score of 90.25%. However, according to the reproduction result, it only obtained the accuracy of 56.67%, precision of 52.92%, sensitivity of 86.00%, AUC of 47.49%, and F1 Score of 64.37%.

### Reproduction results

We have summarized the case study results, including both the original paper’s reported results and our reproduction results. The code of our reproduction can be accessed via: https://github.com/yiming95/PD_ML_benchmark. According to the reproduction, 3 out of 5 papers could not replicate the presented results. Most of the reviewed papers do not provide source code (MRI: 2, voice: 1, handwriting: 1, gait: 2, EEG: 0, and others: 1). The lack of open-source code negatively impacts the understanding and improvement of existing methods. Additionally, even for the studies that provide code, many fail to include complete code, data preprocessing steps, or specific hyperparameter values. These issues have led to many experiments failing to match the original findings.

More specifically, for the voice data modality, we have successfully reproduced the results with 100% accuracy. For the EEG data modality, the original paper reported an accuracy of 98%, whereas our reproduction result is 62.23%. Since the authors did not release their source code, we have re-implemented the model architecture based on the descriptions provided. The discrepancy may be due to the missing description of the implementation details in the original paper, such as the potential use of pre-trained model initialization or specific training techniques that were not disclosed in the original paper. For the gait data modality, the results differ slightly. A possible reason for this could be variations in hyperparameter tuning strategies. The original authors may not have provided the full set of hyperparameters for their model, leading to slight inconsistencies in the reproduced results. For the handwriting data modality, although the authors provided the code, our reproduced results have shown minor discrepancies. A likely explanation is the use of random data splitting, which can result in inconsistent datasets for model training. We believe this discrepancy is due to the absence of exact dataset splits, but it can be reproduced under certain dataset split, and we consider this result is reproducible. For the MRI data modality, the original authors did not release their source code, and key implementation details were also missing from the paper, which could have significantly influenced performance.

As researchers and developers struggle to validate and reproduce previous results, it affects the credibility and transparency of scientific research. Moreover, most of the models lack explainability, which can make health professionals hesitant to trust and adopt these AI tools. Without understanding how the AI system reached its diagnosis, there is a risk of misdiagnosis. If the AI system’s lack of interpretability leads to errors, doctors may find it difficult to identify and correct the issue, which could result in the wrong treatment for patients, severely affecting their health and quality of life. The complete availability of the codes and explainability for all included studies is shown in Table 4.

**Table 3:** Case studies results

Data modality	Paper Report Result	Reproduction Result	Explainability	Robust/Security
Voice	Accuracy: 100%	Accuracy: 100%	Lorenz Zonoid: Cannot be calculated because AUC cannot be calculated	RGR: 99.93%
		Specificity: 0.00%		
		Sensitivity: 100.00%		
		Precision: 100.00%		
		Recall: 100.00%		
		F1 score: 100.00%		
		AUC: Cannot be calculated because there is only one class in the test set		
		RGA: 100.00%		
Gait	Accuracy: 95.2%	Accuracy: 87.12%	Lorenz Zonoid: 69.59%	RGR: 99.84%
	Specificity: 86.8%	Specificity: 68.24%		
	Sensitivity: 98.1%	Sensitivity: 94.03%		
		Precision: 88.99%		
		Recall: 94.03%		
		F1 score: 91.44%		
		AUC: 84.80%		
		RGA: 84.80%		
EEG	Accuracy: 98%	Accuracy: 62.40%	Lorenz Zonoid: 35.60%	RGR: 99.84%
	Specificity: 100%	Specificity: 62.68%		
	Sensitivity: 97%	Sensitivity: 62.10%		
		Precision: 62.00%		
		Recall: 62.10%		
		F1 score: 62.05%		
		AUC: 67.80%		
		RGA: 67.80%		
MRI	Accuracy: 90.36%	Accuracy: 56.67%	Lorenz Zonoid: -5.02%	RGR: 84.31%
	Sensitivity: 90.52%	Specificity: 16.67%		
	Precision: 90.08%	Sensitivity: 86.00%		
	F1 score: 90.25%	Precision: 52.92%		
	AUC: 90.51%	Recall: 86.00%		
		F1 score: 64.37%		
		AUC: 47.49%		
		RGA: 47.49%		
Handwriting	SP_50_50:	SP_50_50:	SP_50_50:	SP_50_50:
	Accuracy: 85.38% (Std: 2.37%)	Accuracy: 85.49% (Std: 2.23%)	Lorenz Zonoid: 87.10% (Std: 2.26%)	RGR: 100.00% (Std: 0.00%)
	Precision: 85.5% (Std: 3.1%)	Specificity: 86.43% (Std: 2.24%)		
	Recall: 83.4% (Std: 5.4%)	Sensitivity: 84.44% (Std: 5.49%)		
	F1 score: 84.3% (Std: 2.9%)	Precision: 84.91% (Std: 1.86%)		
		Recall: 84.44% (Std: 5.49%)		
		F1 score: 84.56% (Std: 2.83%)		
		AUC: 93.55% (Std: 1.13%)		
		RGA: 93.55% (Std: 1.13%)		
	SP_75_25:	SP_75_25:	SP_75_25:	SP_75_25:
	Accuracy: 89.48% (Std: 3.67%)	Accuracy: 84.03% (Std: 2.67%)	Lorenz Zonoid: 88.04% (Std: 3.11%)	RGR: 100.00% (Std: 0.00%)
	Precision: 84.8% (Std: 4.7%)	Specificity: 88.00% (Std: 7.86%)		
	Recall: 95.5% (Std: 4.8%)	Sensitivity: 79.69% (Std: 7.30%)		
	F1 score: 89.7% (Std: 3.5%)	Precision: 86.79% (Std: 6.60%)		
		Recall: 79.69% (Std: 7.30%)		
		F1 score: 82.61% (Std: 2.92%)		
		AUC: 94.02% (Std: 1.55%)		
		RGA: 94.02% (Std: 1.55%)		
	MEA_50_50:	MEA_50_50:	MEA_50_50:	MEA_50_50:
	Accuracy: 89.29% (Std: 3.75%)	Accuracy: 82.03% (Std: 1.92%)	Lorenz Zonoid: 80.62% (Std: 2.29%)	RGR: 100.00% (Std: 0.00%)
	Precision: 85.0% (Std: 4.5%)	Specificity: 83.71% (Std: 5.00%)		
	Recall: 77.9% (Std: 7.9%)	Sensitivity: 80.16% (Std: 5.32%)		
	F1 score: 81.0% (Std: 5.0%)	Precision: 81.96% (Std: 4.05%)		
		Recall: 80.16% (Std: 5.32%)		
		F1 score: 80.81% (Std: 2.27%)		
		AUC: 90.31% (Std: 1.14%)		
		RGA: 90.31% (Std: 1.14%)		
	MEA_75_25:	MEA_75_25:	MEA_75_25:	MEA_75_25:
	Accuracy: 92.24% (Std: 2.65%)	Accuracy: 79.40% (Std: 3.52%)	Lorenz Zonoid: 72.46% (Std: 6.75%)	RGR: 100.00% (Std: 0.00%)
	Precision: 95.2% (Std: 2.5%)	Specificity: 90.57% (Std: 3.63%)		
	Recall: 88.3% (Std: 4.9%)	Sensitivity: 67.19% (Std: 8.76%)		
	F1 score: 92.4% (Std: 3.1%)	Precision: 86.95% (Std: 3.57%)		
		Recall: 67.19% (Std: 8.76%)		
		F1 score: 75.41% (Std: 5.38%)		
		AUC: 86.23% (Std: 3.38%)		
		RGA: 86.23% (Std: 3.38%)		

Std represents the standard deviations. SP_50_50 and SP_75_25 represent experiments using the Spiral Dataset, with 50%/50% and 75%/25% splits for training and testing, respectively. MEA_50_50 and MEA_75_25 represent experiments using the Meander Dataset with the same respective training/testing splits

**Table 4:** Summary of the code availability, data accessibility and explainability for the reviewed paper

Author	Year	Objective	Data Modality	Source Code Provided	Data Accessibility	Explainability
*Neuroimaging*						
Prashanth et al. (2014)	2014	Classification (PD vs. HC)	Neuroimaging: DaTSCAN SPECT	NO	https://www.ppmi-info.org/data	NO
Salvatore et al. (2014)	2014	Classification (PD vs. HC)	Neuroimaging: MRI	NO	NO	NO
Rana et al. (2015)	2015	Classification (PD vs. HC)	Neuroimaging: MRI	NO	NO	NO
Oliveira and Castelo-Branco (2015)	2016	Classification (PD vs. HC)	Neuroimaging: FP-CIT SPECT	NO	https://www.ppmi-info.org/data	NO
Zhang and Kagen (2017)	2017	Classification (PD vs. HC)	Neuroimaging: DaTSCAN SPECT	NO	https://www.ppmi-info.org/data	NO
Peng et al. (2017)	2017	Classification (PD vs. HC)	Neuroimaging: MRI	NO	https://www.ppmi-info.org/data	NO
Sivaranjini and Sujatha (2020)	2020	Classification (PD vs. HC)	Neuroimaging: MRI	NO	https://www.ppmi-info.org/data	NO
West et al. (2019)	2019	Classification (PD vs. HC)	Neuroimaging: MRI	NO	https://www.ppmi-info.org/data	NO
Dai et al. (2019)	2019	Classification (PD vs. HC)	Neuroimaging: PET	NO	https://www.ppmi-info.org/data; https://adni.loni.usc.edu/; https://db.humanconnectome.org/app/template/Login.vm	NO
Zhang et al. (2019)	2019	Classification (Prodromal PD vs. Confirmed PD vs. HC)	Neuroimaging: MRI	NO	https://www.ppmi-info.org/data	NO
Chakraborty et al. (2020)	2020	Classification (PD vs. HC)	Neuroimaging: MRI	NO	https://www.ppmi-info.org/data	NO
Kaur et al. (2021)	2021	Classification (PD vs. HC)	Neuroimaging: MRI	NO	https://www.ppmi-info.org/data	NO
Vyas et al. (2022)	2022	Classification (PD vs. HC)	Neuroimaging: MRI	NO	https://www.ppmi-info.org/data	NO
Ya et al. (2022)	2022	Classification (PD vs. NC)	Neuroimaging: MRI	NO	NO	NO
Erdaş and Sümer (2022)	2022	Classification (PD vs. NC)	Neuroimaging: MRI	NO	https://fcon_1000.projects.nitrc.org/indi/retro/parkinsons.html	NO
Huang et al. (2023)	2023	Classification (PD vs. HC)	Neuroimaging: MRI	https://gitee.com/yxfamy/mnc-net_master.git (Currently 403 cannot access)		YES
Xu et al. (2023)	2023	Classification (PD vs. HC)	Neuroimaging: MRI	https://github.com/ymlasu/A-Bio-marker-using-Topological-Machine-Learning-of-rs-fMRI (Only part of the code is provided)	https://www.ppmi-info.org/data	NO
Camacho et al. (2023)	2023	Classification (PD vs. HC)	Neuroimaging: MRI	NO	https://www.ppmi-info.org/data	YES
Priyadharshini et al. (2024)	2024	Classification (PD vs. HC)	Neuroimaging: 3D MRI	NO		YES
Talai et al. (2021)	2021	Classification (PD vs. PSP vs. HC)	Neuroimaging: T1, T2, DTI MRI	NO	https://www.ppmi-info.org/data	NO
Prasuhn et al. (2020)	2020	Classification (PD vs. HC)	Neuroimaging: Diffusion Tensor Imaging (DTI)	NO	https://www.ppmi-info.org/data	NO
Chen et al. (2023)	2023	Classification (PD-MCI vs. PD-NC)	Neuroimaging: DTI (FA, MD, AD, RD, LDH)	NO	contact corresponding author for access	YES
Tsai et al. (2023)	2023	Classification (PD vs. PSP vs. MSA vs. HC)	Neuroimaging: DTI (whole-brain features)	NO	NO	NO
Zhao et al. (2022)	2022	Classification (PD vs. HC)	Neuroimaging: DTI (Fractional Anisotropy, MD)	NO	NO	NO
*Voice*						
Sakar and Kursun (2010)	2010	Classification (PD vs. HC)	Voice dataset	NO	https://archive.ics.uci.edu/dataset/174/parkinsons	NO
Bhattacharya and Bhatia (2010)	2010	Classification (PD vs. HC)	Voice dataset	https://www.csie.ntu.edu.tw/~cjlin/libsvm/ (Only part of the code is provided)	https://archive.ics.uci.edu/dataset/174/parkinsons	NO
Guo et al. (2010)	2010	Classification (PD vs. HC)	Voice dataset	NO	https://archive.ics.uci.edu/dataset/174/parkinsons	NO
Åström and Koker (2011)	2011	Classification (PD vs. HC)	Voice dataset	NO	https://archive.ics.uci.edu/dataset/174/parkinsons	NO
Ramani and Sivagami (2011)	2011	Classification (PD vs. HC)	Voice dataset	NO	https://archive.ics.uci.edu/dataset/174/parkinsons	NO
Yadav et al. (2012)	2012	Classification (PD vs. HC)	Voice dataset	NO	https://archive.ics.uci.edu/dataset/174/parkinsons	NO
Tsanas et al. (2012)	2012	Classification (PD vs. HC)	Voice dataset	NO	NO	NO
Mandal and Sairam (2014)	2014	Classification (PD vs. HC)	Voice dataset	NO	https://archive.ics.uci.edu/dataset/174/parkinsons	NO
Hazan et al. (2012)	2012	Classification (PD vs. HC)	Voice dataset	NO	NO	NO
Gharehchopogh and Mohammadi (2013)	2013	Classification (PD vs. HC)	Voice dataset	NO	https://archive.ics.uci.edu/dataset/174/parkinsons	NO
Rustempasic and Can (2013)	2013	Classification (PD vs. HC)	Voice dataset	NO	https://archive.ics.uci.edu/dataset/174/parkinsons	NO
Sharma and Giri (2014)	2014	Classification (PD vs. HC)	Voice dataset	NO	https://archive.ics.uci.edu/dataset/174/parkinsons	NO
Olanrewaju et al. (2014)	2014	Classification (PD vs. HC)	Voice dataset	NO	https://archive.ics.uci.edu/dataset/174/parkinsons	NO
Peker et al. (2015)	2015	Classification (PD vs. HC)	Voice dataset	NO	https://archive.ics.uci.edu/dataset/174/parkinsons	NO
Gök (2015)	2015	Classification (PD vs. HC)	Voice dataset	NO	https://archive.ics.uci.edu/dataset/174/parkinsons	NO
Chen et al. (2016)	2016	Classification (PD vs. HC)	Voice dataset	NO	https://archive.ics.uci.edu/dataset/174/parkinsons	NO
Avci and Dogantekin (2016)	2016	Classification (PD vs. HC)	Voice dataset	NO	https://archive.ics.uci.edu/dataset/174/parkinsons	NO
Dinesh and He (2017)	2017	Classification (PD vs. HC)	Voice dataset	NO	https://archive.ics.uci.edu/dataset/174/parkinsons	NO
Caliskan et al. (2017)	2017	Classification (PD vs. HC)	Voice dataset	NO	https://archive.ics.uci.edu/dataset/174/parkinsons	NO
Parisi et al. (2018)	2018	Classification (PD vs. HC)	Voice dataset	NO	https://archive.ics.uci.edu/dataset/301/parkinson+speech+dataset+with+multiple+types+of+sound+recordings	NO
Wroge et al. (2018)	2018	Classification (PD vs. HC)	Voice dataset	NO	NO	NO
Lahmiri et al. (2018)	2018	Classification (PD vs. HC)	Voice dataset	NO	NO	NO
Haq et al. (2018)	2018	Classification (PD vs. HC)	Voice dataset	NO	https://archive.ics.uci.edu/dataset/174/parkinsons	NO
Ali et al. (2019)	2019	Classification (PD vs. HC)	Voice dataset	https://github.com/LiaqatAli007/Automated-Detection-of-Parkinson-s-Disease-Based-on-Multiple-Types-of-Sustained-Phonations-using-Lin	https://archive.ics.uci.edu/dataset/301/parkinson+speech+dataset+with+multiple+types+of+sound+recordings	NO
Mostafa et al. (2019)	2019	Classification (PD vs. HC)	Voice dataset	NO	https://archive.ics.uci.edu/dataset/174/parkinsons	NO
Lahmiri and Shmuel (2019)	2019	Classification (PD vs. HC)	Voice dataset	NO	NO	NO
Haq et al. (2019)	2019	Classification (PD vs. HC)	Voice dataset	NO	https://archive.ics.uci.edu/dataset/174/parkinsons	NO
Senturk (2020)	2020	Classification (PD vs. HC)	Voice dataset	NO	https://archive.ics.uci.edu/dataset/174/parkinsons	NO
Karan et al. (2020)	2020	Classification (PD vs. HC)	Voice dataset	NO	NO	NO
Soumaya et al. (2021)	2021	Classification (PD vs. HC)	Voice dataset	NO	NO	NO
Karaman et al. (2021)	2021	Classification (PD vs. HC)	Voice dataset	NO	NO	NO
Quan et al. (2021)	2021	Classification (PD vs. HC)	Voice dataset	NO	NO	NO
Zahid et al. (2020)	2020	Classification (PD vs. HC)	Voice dataset	NO	NO	NO
Rizvi et al. (2020)	2020	Classification (PD vs. HC)	Voice dataset	NO	https://archive.ics.uci.edu/dataset/301/parkinson+speech+dataset+with+multiple+types+of+sound+recordings	NO
Abayomi-Alli et al. (2020)	2020	Classification (PD vs. HC)	Voice dataset	NO	https://archive.ics.uci.edu/dataset/174/parkinsons	NO
Gunduz (2019)	2019	Classification (PD vs. HC)	Voice dataset	NO	https://archive.ics.uci.edu/dataset/470/parkinson+s+disease+classification	NO
Nagasubramanian and Sankayya (2021)	2021	Classification (PD vs. HC)	Voice dataset	NO	NO	NO
Fang et al. (2020)	2020	Classification (PD vs. HC)	Voice dataset	NO	NO	NO
Ali et al. (2023)	2023	Classification (PD vs. HC)	Voice dataset	NO	NO	NO
Hireš et al. (2022)	2022	Classification (PD vs. HC)	Voice dataset	NO	NO	NO
Rana et al. (2022)	2022	Classification (PD vs. HC)	Voice dataset	NO	Avaiable on Request	NO
Madruga et al. (2023)	2023	Classification (PD vs. HC)	Voice dataset	NO	NO	NO
Govindu and Palwe (2023)	2023	Classification (PD vs. HC)	Voice dataset	NO	https://archive.ics.uci.edu/dataset/174/parkinsons	NO
Celik and Başaran (2023)	2023	Classification (PD vs. HC)	Voice dataset	NO	https://archive.ics.uci.edu/dataset/174/parkinsons;https://archive.ics.uci.edu/dataset/470/parkinson+s+disease+classification	NO
Khaskhoussy and Ayed (2023)	2023	Classification (PD vs. HC)	Voice dataset	NO	https://archive.ics.uci.edu/dataset/301/parkinson+speech+dataset+with+multiple+types+of+sound+recordings	NO
Dheer et al. (2023)	2023	Classification (PD vs. HC)	Voice dataset	NO	https://archive.ics.uci.edu/dataset/174/parkinsons	NO
Akila and Nayahi (2024)	2024	Classification (PD vs. HC)	Voice dataset	NO	https://archive.ics.uci.edu/dataset/470/parkinson+s+disease+classification	NO
*Handwriting*						
Drotár et al. (2014)	2014	Classification (PD vs. HC)	Handwriting dataset	NO	NO	NO
Drotár et al. (2015)	2015	Classification (PD vs. HC)	Handwriting dataset	NO	NO	NO
Pereira et al. (2015)	2015	Classification (PD vs. HC)	Handwriting dataset	NO	NO	NO
Ribeiro et al. (2019)	2019	Classification (PD vs. HC)	Handwriting dataset	https://github.com/lzfelix/bag-of-samplings	https://wwwp.fc.unesp.br/~papa/pub/datasets/Handpd/	NO
Razzak et al. (2020)	2020	Classification (PD vs. HC)	Handwriting dataset	NO	https://wwwp.fc.unesp.br/~papa/pub/datasets/Handpd/;https://www.kaggle.com/datasets/kmader/parkinsons-drawings; https://bdalab.utko.fekt.vut.cz/	NO
Kamran et al. (2021)	2021	Classification (PD vs. HC)	Handwriting dataset	NO	https://wwwp.fc.unesp.br/~papa/pub/datasets/Handpd/;https://www.kaggle.com/datasets/kmader/parkinsons-drawings; https://bdalab.utko.fekt.vut.cz/	NO
Gil-Martín et al. (2019)	2019	Classification (PD vs. HC)	Handwriting dataset	NO	https://archive.ics.uci.edu/dataset/395/parkinson+disease+spiral+drawings+using+digitized+graphics+tablet	NO
Diaz et al. (2021)	2021	Classification (PD vs. HC)	Handwriting dataset	NO	https://wwwp.fc.unesp.br/~papa/pub/datasets/Handpd/	NO
Taleb et al. (2019)	2019	Classification (PD vs. HC)	Handwriting dataset	NO	https://wwwp.fc.unesp.br/~papa/pub/datasets/Handpd/	NO
Varalakshmi et al. (2022)	2022	Classification (PD vs. HC)	Handwriting dataset	NO	https://www.kaggle.com/datasets/kmader/parkinsons-drawings	NO
Li et al. (2022)	2022	Classification (PD vs. HC)	Handwriting dataset	NO	NO	NO
Zhao and Li (2023)	2023	Classification (PD vs. HC)	Handwriting dataset	NO	https://wwwp.fc.unesp.br/~papa/pub/datasets/Handpd/	NO
Abdullah et al. (2023)	2023	Classification (PD vs. HC)	Handwriting dataset	NO	https://wwwp.fc.unesp.br/~papa/pub/datasets/Handpd/	NO
Wang et al. (2024)	2024	Classification (PD vs. HC)	Handwriting dataset	NO	NO	NO
*Gait*						
Tahir and Manap (2012)	2012	Classification (PD vs. HC)	Gait dataset	NO	NO	NO
Wahid et al. (2015)	2015	Classification (PD vs. HC)	Gait dataset	NO	NO	NO
Shetty and Rao (2016)	2016	Classification (PD vs. HD vs. ALS)	Gait dataset	NO	https://physionet.org/content/gaitpdb/1.0.0/	NO
Abdulhay et al. (2018)	2018	Classification (PD vs. HC)	Gait dataset	NO	https://physionet.org/content/gaitpdb/1.0.0/	NO
Rehman et al. (2019)	2019	Classification (PD vs. HC)	Gait dataset	NO	NO	NO
Balaji et al. (2021)	2021	Classification (PD vs. HC)	Gait dataset	NO	https://physionet.org/content/gaitpdb/1.0.0/	NO
Xia et al. (2019)	2019	Classification (PD vs. HC)	Gait dataset	NO	https://physionet.org/content/gaitpdb/1.0.0/	NO
El Maachi et al. (2020)	2020	Classification (PD vs. HC)	Gait dataset	NO	https://physionet.org/content/gaitpdb/1.0.0/	NO
Aversano et al. (2020)	2020	Classification (PD vs. HC)	Gait dataset	NO	https://physionet.org/content/gaitpdb/1.0.0/	NO
Liu et al. (2021)	2021	Classification (PD vs. HC)	Gait dataset	Submit an application to the author	https://physionet.org/content/gaitpdb/1.0.0/	NO
Nguyen et al. (2022)	2022	Classification (PD vs. HC)	Gait dataset	https://github.com/DucMinhDimitriNguyen	https://physionet.org/content/gaitpdb/1.0.0/	NO
Trabassi et al. (2022)	2022	Classification (PD vs. HC)	Gait dataset	NO	Request from the corresponding author	NO
Li and Li (2022)	2022	Classification (PD vs. HC)	Gait dataset	NO	https://physionet.org/content/gaitpdb/1.0.0/	NO
Aşuroğlu and Oğul (2022)	2022	Classification (PD vs. HC), Regression (UPDRS value)	Gait dataset	NO	https://physionet.org/content/gaitpdb/1.0.0/	NO
Ma et al. (2023)	2023	Classification (PD vs. HC)	Gait dataset	NO	https://physionet.org/content/gaitpdb/1.0.0/	NO
Vinora et al. (2023)	2023	Classification (PD vs. HC)	Gait dataset	NO	NO	NO
Sharma et al. (2023)	2023	Classification (PD vs. HC)	Gait dataset	NO	https://physionet.org/content/gaitpdb/1.0.0/	NO
*EEG*						
Lee et al. (2019)	2019	Classification (PD vs. HC)	EEG	NO	NO	NO
Oh et al. (2020)	2020	Classification (PD vs. HC)	EEG	NO	NO	NO
Anjum et al. (2020)	2020	Classification (PD vs. HC)	EEG	NO	http://narayanan.lab.uiowa.edu/;http://predict.cs.unm.edu/	NO
Shaban (2021)	2021	Classification (PD vs. HC)	EEG	NO	https://openneuro.org/datasets/ds002778/versions/1.0.5	NO
Loh et al. (2021)	2021	Classification (PD vs. HC)	EEG	NO	https://openneuro.org/datasets/ds002778/versions/1.0.5	NO
Motin et al. (2022)	2022	Classification (PD vs. HC)	EEG	NO	https://openneuro.org/datasets/ds002778/versions/1.0.5	YES
Chawla et al. (2023)	2023	Classification (PD vs. HC)	EEG	NO	NO	NO
Coelho et al. (2023)	2023	Classification (PD vs. HC)	EEG	NO	http://predict.cs.unm.edu/	NO
Nour et al. (2023)	2023	Classification (PD vs. HC)	EEG	NO	https://openneuro.org/datasets/ds002778/versions/1.0.5	NO
Zhao et al. (2024)	2024	Classification (PD vs. HC)	EEG	Request from the corresponding author	NO	NO
*Other Data*						
Bhandari et al. (2023)	2023	Classification (PD vs. HC)	Gene dataset	https://github.com/nikitabhandari-dl/Parkinson-s-disease-diagnosis (Currently 404 cannot access)	https://ngdc.cncb.ac.cn/	YES
Wang et al. (2023)	2023	Classification (PD vs. HC)	Urine biomarkers	NO	NO	NO
Junaid et al. (2023)	2023	Classification (PD vs. HC)	Patient visits	NO	https://www.ppmi-info.org/	YES
Igene et al. (2023)	2023	Classification (PD vs. HC)	Movement data	NO	https://doi.org/10.21227/g2g8-1503	NO
Varghese et al. (2024)	2024	Classification (PD vs. HC)	Smartwatch data, Questionnaire data	https://imigitlab.uni-muenster.de/published/pads-project	https://uni-muenster.sciebo.de/s/q69vUfRc9vgBoWX	NO

## Discussions

### Summary of findings

**Fig. 12:**
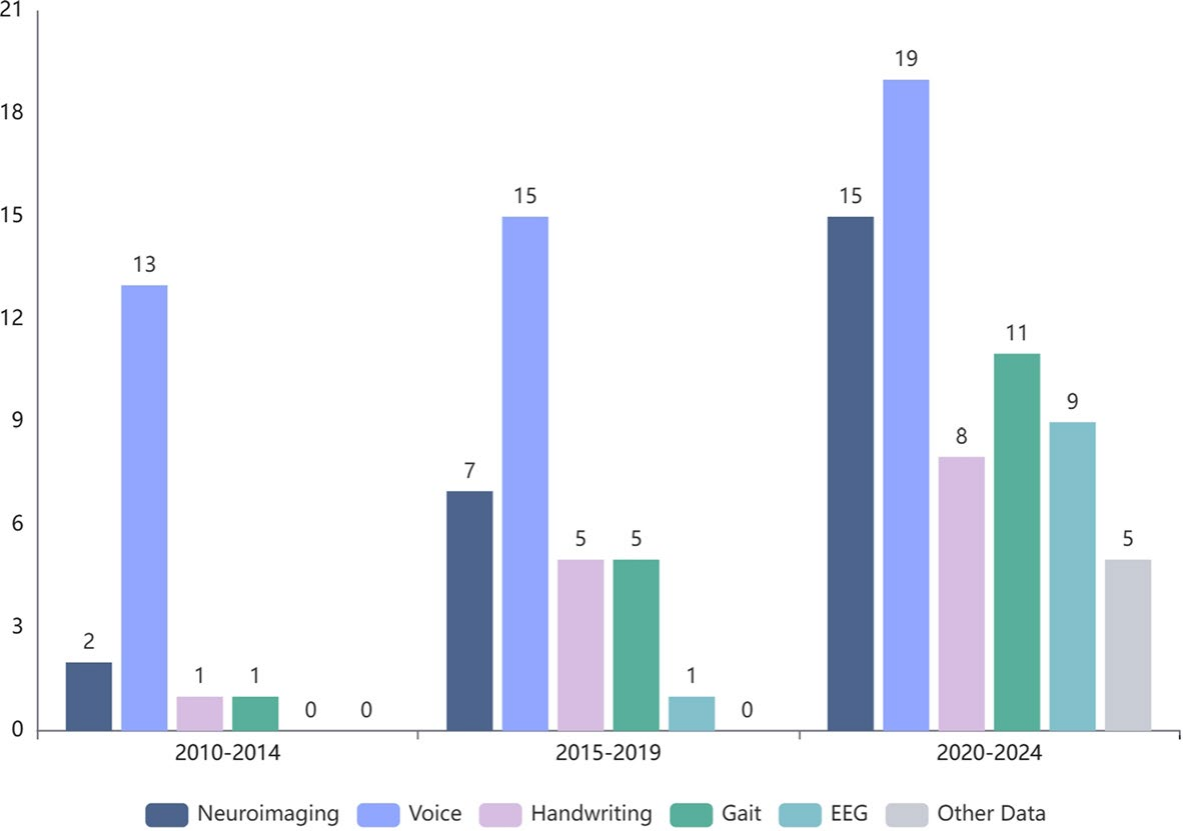
The development and changes of data in different modalities

ML-based PD diagnosis is a rapidly growing and changing field of research. This systematic review includes 117 articles about PD diagnosis using ML from 2010 to 2024. We analyze and divide them into six categories based on the data modality used in the study: (1) Neuroimaging, (2) Voice, (3) Handwriting, (4) Gait, (5) EEG, and (6) Other data. Fig 12. provides the trends of the publication for the last 15 years (2010-2024). Compared with other modalities, the neuroimaging modality, especially DaTSCAN SPECT, is the best modality for PD diagnosis in clinical practice, whereas MRI is almost useless. However, the usage of neuroimaging can be expensive. Voice recording, handwriting, and gait data are non-invasive, cost-effective, and easily collected. Hence, these data may be used for PD diagnosis. The main disadvantage of using these modalities is the lack of uniform standards for data collection, which may lead to inaccurate diagnosis. In clinical practice, EEG is not useful for the diagnosis of PD. However, a few studies have used EEG to diagnose PD, and the validity of this modality needs to be further investigated by researchers in this field.

**Fig. 13:**
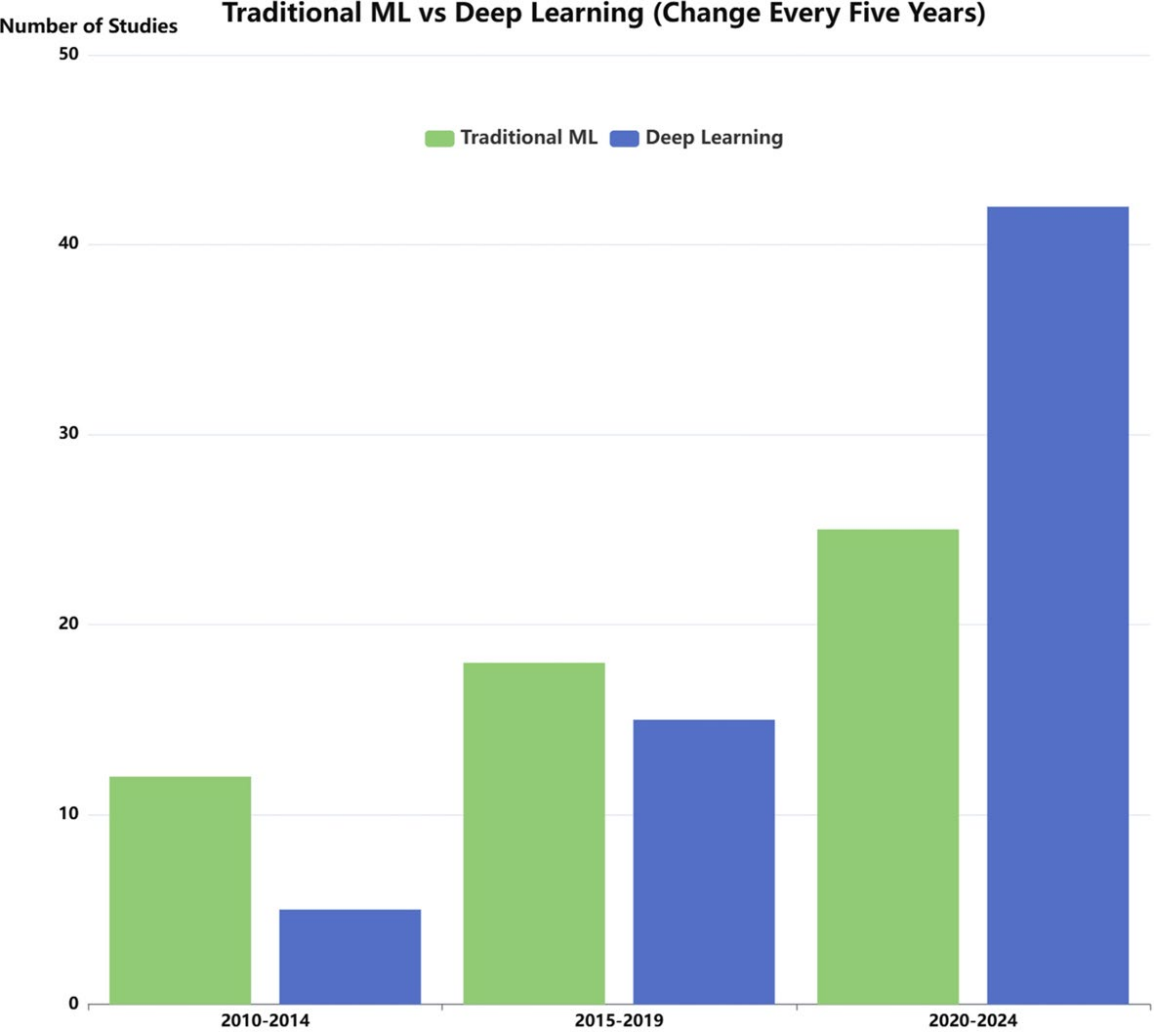
Temporal evolution of the application of traditional ML and DL techniques in PD classification research over five-year intervals from 2010 to 2024

We have also summarized the changes in the application of traditional ML and DL in PD diagnosis over the past 15 years. Fig 13 illustrates the temporal evolution of the application of traditional ML and DL techniques in PD classification research over five-year intervals from 2010 to 2024. During the early years from 2010 to 2014, traditional ML methods such as SVM and Random Forest dominated the field, while there was less use of the DL approach. During the period from 2015 to 2019, DL gained momentum and nearly caught up with traditional ML methods. A major shift occurred in the period from 2020 to 2024, where the number of studies employing DL significantly surpassed those using traditional ML, which has established DL as the mainstream approach. This trend reflects the increasing availability of large-scale datasets, advancements in computational resources, and the superior performance of deep neural networks in complex biomedical classification tasks.

Across the 117 studies reviewed in our systematic review, the main issue is that comparing the model performance with different modalities is hard. For example, the clinical value for an ML-based PD diagnosis using neuroimaging and voice recordings differs. Another issue is that the authors needed to provide more implementation details. For instance, some articles have not reported hyperparameters clearly, which may cause difficulty in reproducing the experiments. In addition, some articles only used accuracy as the evaluation metric of the model, which is not reasonable. Accuracy can be misleading when the data is imbalanced, meaning that there are significantly more samples of one class than the others. Also, different misclassification errors can have different costs in real-world applications. In a medical diagnosis task, a false negative (i.e., a patient is predicted as not having a disease when they do) can have severe consequences compared to a false positive (i.e., a patient is predicted as having a disease when they don’t). Therefore, more evaluation metrics such as specificity and sensitivity should be considered.

### Limitations of current studies

#### Dataset size

This review has identified several limitations of existing studies that have applied ML to PD diagnosis. Firstly, the number of database participants with PD is often relatively small; for example, the total number of subjects may be less than 50 (Sakar and Kursun 2010; Bhattacharya and Bhatia 2010; Guo et al. 2010; Åström and Koker 2011; Ramani and Sivagami 2011; Yadav et al. 2012; Mandal and Sairam 2014; Gharehchopogh and Mohammadi 2013; Rustempasic and Can 2013; Sharma and Giri 2014; Olanrewaju et al. 2014; Peker et al. 2015; Gök 2015; Chen et al. 2016; Avci and Dogantekin 2016; Dinesh and He 2017; Caliskan et al. 2017; Parisi et al. 2018; Haq et al. 2018; Ali et al. 2019; Mostafa et al. 2019; Lahmiri and Shmuel 2019; Haq et al. 2019; Senturk 2020; Soumaya et al. 2021; Quan et al. 2021; Rizvi et al. 2020; Abayomi-Alli et al. 2020; Govindu and Palwe 2023; Khaskhoussy and Ayed 2023; Dheer et al. 2023; Ribeiro et al. 2019; Taleb et al. 2019; Tahir and Manap 2012; Wahid et al. 2015; Shetty and Rao 2016; Oh et al. 2020; Shaban 2021; Loh et al. 2021; Motin et al. 2022; Chawla et al.^2023^; Igene et al. 2023). Only eight included articles have over 500 number of subjects (Prashanth et al. 2014; Oliveira and Castelo-Branco 2015; Zhang et al. 2019; Camacho et al. 2023; Priyadharshini et al. 2024; Tsai et al. 2023; Zhao et al. 2022; Bhandari et al. 2023). The small data size may limit the performance of the ML models.

#### Black box nature of ML models

Another challenge is the black-box nature of the ML model, which limits the clinical applications of ML in PD diagnosis. ML algorithms, such as SVM and DL models such as CNN and RNN, are all examples of black-box models. These models contain a large number of parameters, making it difficult to interpret how they arrive at their decisions. This makes it challenging to understand why a particular diagnosis is being made, and this lack of transparency can be a significant barrier to the adoption of ML in clinical environments. The diagnosis of PD is a life safety-critical medical task, where the accuracy of diagnosis is essential for the patient’s treatment and management. Therefore, there is a need to not only use ML as a decision-support tool but also to ensure that the ML models used are interpretable to medical experts and patients. Interpretable ML models allow doctors and patients to understand the reasoning behind the model’s decision-making process, thereby increasing their trust in the model’s accuracy and reliability. Interpretable ML models provide insights into the input features that have the most significant impact on the diagnosis, the relationship between the input features and the output, and how the model arrives at its final decision.

#### No standardization of validation

This review has identified a lack of standardization of validation. Included studies used different validation methods, including k-fold cross-validation and hold-out validation. The use of different validation methods makes comparisons between different studies difficult. More specifically, if one study claims that it outperformed the state-of-the-art (SOTA), the proposed methodology should at least replicate other SOTA methods under the same dataset, same experiment setup and exact validation mechanism. Otherwise, it is unconvincing, as the dataset’s bias and validation mechanism may produce this better performance and not necessarily the ML algorithm design.

#### Lack of medical experts’ participation

Most studies follow a typical sequence. First, different modality data are collected and processed from PD and healthy control participants. Next, clinical experts manually annotate the dataset. Finally, the ML model is trained to classify patients and healthy controls. Thus, clinicians only contribute to the data label annotation, which limits the performance of the ML model building. ML scientists and medical experts should collaborate at all stages to provide feedback on the model performance and give valuable suggestions on model selection and explanation.

#### Bias Risk and Trustworthiness of ML-Based PD Diagnosis

Despite the growing body of ML research on PD diagnosis, only 28 out of the 117 reviewed studies have been assessed as having an overall low risk of bias based on our PROBAST evaluation. Common issues include small sample sizes, lack of external validation, unclear blinding procedures, and potential data leakage during feature selection. These limitations significantly impact the reliability and generalizability of ML models. A model that performs well within a single cohort may still fail when applied to external or real-world clinical settings. Thus, confidence in ML-based diagnostic tools depends not only on predictive performance but also on methodological rigor and transparency. High-risk bias compromises both reproducibility and the level of clinical trust necessary for real-world deployment.

#### No standardizing ML approaches

Our systematic review reveals that there is currently no standardized ML approach for the diagnosis of PD. One of the key obstacles to achieving generalizable and reproducible ML models is the lack of standardization across publicly available datasets. This issue significantly hinders fair model comparison, reproducibility, and clinical translation. First, there is considerable heterogeneity in data acquisition protocols. Different datasets are collected using varying configurations; for example, EEG sampling rates may differ (e.g., 128 Hz vs. 1024 Hz), MRI scans may be acquired using different field strengths (e.g., 1.5T vs. 3T), and voice recordings may be captured under inconsistent environmental conditions. These discrepancies lead to variations in signal quality and frequency content, which directly affect feature extraction and model performance. Second, substantial variability exists in patient cohorts and diagnostic labelling. Datasets differ in inclusion criteria (e.g., drug-naïve vs. medicated patients), disease stage distributions, age ranges, and definitions of control groups. Furthermore, diagnostic labels are often assigned based on different clinical criteria, such as the MDS-UPDRS, Hoehn and Yahr staging, or clinician judgment, leading to label inconsistency and reduced comparability. Third, inconsistencies in preprocessing and feature engineering pipelines further complicate model standardization. Many studies employ custom workflows, such as filtering, artifact removal, and dimensionality reduction, that are often poorly documented and difficult to reproduce. In some cases, parameter tuning may even occur on the test set, introducing additional bias into performance evaluation. Finally, differences in data modalities and formats add to the complexity. Multimodal datasets often vary in terms of synchronization and alignment between modalities. Some datasets provide only raw signals, while others include derived features or lack essential metadata, making it challenging to develop standardized multimodal fusion methods.

### Future research directions

#### Explainable artificial intelligence (XAI)

XAI aims to provide understandable human explanations to users to better understand the black box models’ decision process (Zhang et al. 2022). The XAI approach has the potential to generate improved models and verified predictions. Moreover, an XAI system can help clinicians and researchers to understand the reasoning behind an AI system’s decision and to identify potential biases or limitations in the model. This can help to improve the accuracy and reliability of PD diagnosis, which can have important implications for patient outcomes.

#### Data augmentation

Data augmentation is a method to generate synthetic data. As the dataset size used for ML-based PD diagnosis is relatively small, data augmentation is a feasible approach to increase the dataset size and further improve the performance and the generalisation of the ML model. Different data modalities need to apply different data augmentation methods. Generative Adversarial Networks (GAN) is a promising method which has mostly been applied to generating image data (Yi et al. 2019). It can create diverse and realistic synthetic data that can capture the underlying data distribution, which reduces overfitting in ML models by increasing the diversity of the training data. In the future, using GAN to generate voice, neuroimaging, handwriting, gait, and EEG data for PD diagnosis is also achievable.

#### Transfer learning

The size of the dataset currently used to diagnose PD is insufficient for ML; therefore, transfer learning could be an effective approach to improve training efficiency and speed. When working with a small dataset, there is a higher risk of overfitting, where the model becomes too specialized in the training data and performs poorly on unseen data. To address this issue, transfer learning can be employed by leveraging a pre-trained model that has learned features from a large dataset and transferring that knowledge to a smaller dataset. Additionally, transfer learning can save valuable time and computational resources by reducing the amount of training required for a new model. Instead of training a model from scratch, transfer learning enables the fine-tuning of an existing model on a small dataset, which is a more efficient and quicker process (Kaur et al. 2021).

#### Federated learning

ML models often require large amounts of user data. However, collecting data for PD poses challenges since individual hospitals and organisations collect data, and data sharing may be hindered. Federated learning presents a potential solution for developing models that identify PD biomarkers and patterns using data from various sources, such as medical records, clinical studies, and wearable devices. With federated learning, different parties can collaborate to create a shared model without sharing their data. This approach also facilitates the use of large datasets without centralising the data, which is essential when working with sensitive patient information. Instead, data remains on local devices, and the model is trained by aggregating information across multiple devices without transferring data. Federated learning thus protects patient privacy while enabling the development of accurate models (Rieke et al. 2020).

#### Multi-modality

The multi-modality approach is a promising direction, as it can integrate multiple-view information and perform better than a single modality (Makarious et al. 2022). Single-modality learning is prone to overfitting, especially when the data samples are limited, which is often the case with PD datasets that are small and prone to noise. By incorporating additional modalities, such as genetic analysis, neuroimaging, or EEG, the model can compensate for the lack of data, enhancing its ability to learn from different types of information, thereby improving diagnostic accuracy. Furthermore, the clinical manifestations of PD vary across patients, and a single modality may fail to capture these differences comprehensively. Multi-modal data can ensure that the model generalises better across different patient groups. Additionally, multi-modal models remain robust even when one modality’s data is missing or of poor quality, making reliable predictions without being affected by the absence of any single modality. However, the acquisition of diverse data presents challenges, particularly in data availability, quality, and integration. Developing datasets specifically designed for multi-modal research remains a significant hurdle, and the standardization of data collection protocols across modalities is necessary to ensure consistency. In the future, integrating diverse modalities such as genetic data, blood samples, neuroimaging, voice, handwriting, gait analysis, and EEG into a unified ML framework could significantly improve PD diagnosis, leading to earlier and more accurate diagnoses, better patient stratification, and personalized treatments, ultimately enhancing patient outcomes.

#### Open source culture and standard protocols

To promote the development of ML in the diagnosis of PD, researchers should proactively disclose the full source code and experimental details used in their studies. This includes all necessary steps of data preprocessing, model evaluation, hyperparameter tuning, and pre-trained model. It ensures that other researchers can accurately reproduce and validate experimental results. Additionally, researchers should create standardized datasets collection and evaluation protocols, allowing all methods to be assessed and compared on a fair and uniform basis. At the same time, academic journals should implement stricter peer-review processes, particularly focusing on the reproducibility of the submitted works. Reviewers need to be specifically trained to ensure they can thoroughly assess whether the provided materials are sufficient to replicate the study results. By taking these measures, the transparency and reliability of research can be enhanced, facilitating scientific progress and technological innovation in the field.

#### Ethical concerns

Ethics are important in applying ML and DL to PD diagnosis. First, data privacy and security are major issues, especially in the medical field, where patient health data contains sensitive information. Unauthorised collection and use of data may lead to privacy breaches and even malicious exploitation. Secondly, fairness is a critical concern. If training data is biased, the model may produce inaccurate diagnostic results for certain groups (e.g., specific ages, genders, or ethnicities), exacerbating health inequalities. Moreover, DL models are often seen as “black boxes”, lacking transparency in their decision-making processes. Medical professionals may be hesitant to trust and adopt AI systems if they cannot understand how diagnoses are made. Finally, as AI becomes more integrated into healthcare, the issue of accountability becomes increasingly complex. If an AI system makes a wrong diagnosis leading to harm, who should take responsibility? The developers, the healthcare institution, or the AI itself? These issues need to be carefully addressed within an ethical framework. Future research should focus on developing methods to enhance the interpretability and transparency of AI systems, establishing guidelines for data privacy and security, and creating clear accountability structures. Collaborative efforts between AI researchers, healthcare professionals, and ethicists are essential to ensure that these technologies are implemented responsibly and fairly, mitigating potential risks and improving patient outcomes.

#### More complete model evaluation

In future research, it is important not to rely solely on traditional evaluation metrics such as accuracy, AUC, and precision. While these metrics are undoubtedly valuable, they may not fully capture model performance, particularly in tasks involving ordinal or continuous outcomes. To address this limitation, we advocate for the complementary use of more agnostic and unified evaluation measures, such as the Rank Graduation Accuracy (RGA) proposed by Giudici and Raffinetti (2025). RGA is applicable across binary, ordinal, and continuous predictive settings, offering a more generalizable and consistent framework for comparing models under diverse data conditions and outcome types. Incorporating such metrics alongside traditional ones could significantly improve the fairness, robustness, and clinical relevance of performance evaluation in ML-based disease diagnosis.

Beyond predictive performance, model interpretability is another critical yet often underemphasized component of diagnostic model evaluation. Traditional metrics such as accuracy and AUC provide insights into how well a model performs, but offer little information about why it makes certain predictions. In medical applications, particularly in the diagnosis of complex neurodegenerative diseases such as PD, understanding the rationale behind model decisions is essential for building clinical trust, ensuring transparency, and facilitating adoption in practice. Despite its importance, explainability remains underexplored in many published studies. As shown in the Table. 4, only a limited number of works incorporate explainability techniques, and among those that there is little consistency in the methods used. Furthermore, the lack of open-source implementations prevents systematic comparison across models. To address these gaps, we encourage future research to integrate interpretability as a core component of model development and validation. In particular, model-agnostic explainability methods, which can be applied regardless of the underlying algorithm, should be prioritized, as they enable fairer and more standardized comparisons (Calzarossa et al. 2025). The adoption of such frameworks may also facilitate the identification of clinically relevant biomarkers, thereby strengthening the link between computational models and real-world medical applications.

In addition to performance and interpretability, robustness and security represent two further dimensions that are essential for the safe deployment of diagnostic models but are frequently overlooked. Robustness refers to a model’s ability to maintain stable performance when faced with noise, missing data, or domain shifts — all of which are common in real-world clinical settings. Security, by contrast, concerns the model’s resistance to adversarial examples or malicious attacks that could compromise its output. These aspects are rarely evaluated in existing studies, often due to a lack of reproducibility and the absence of standardized assessment tools. The SAFE AI framework (Babaei et al. 2025), for example, introduces the Rank Graduation Box as a structured, model-agnostic approach to evaluating robustness and security. We therefore recommend that future research explicitly incorporate robustness and security testing into the model evaluation process. Doing so will be crucial for developing trustworthy and clinically deployable AI systems, particularly in high-stakes domains such as healthcare.

#### Bias mitigation

To improve the reliability and clinical applicability of ML models for PD diagnosis, future research must systematically address the sources of bias identified by tools such as PROBAST. It includes implementing rigorous dataset selection with transparent inclusion and exclusion criteria, clearly reporting participant selection logic, and accounting for demographic diversity such as age, disease stage, and comorbidities. Feature selection should be strictly separated from model evaluation to prevent information leakage, an issue commonly caused by selecting features on the entire dataset prior to train-test splitting. Employing nested cross-validation can help mitigate this risk. External validation using independent datasets from different geographic, demographic, or temporal contexts remains essential for demonstrating model generalizability, yet is still underutilized. Moreover, we encourage researchers to explicitly report how each PROBAST domain is addressed, either in the methods section or supplementary materials, to enhance transparency and facilitate cross-study comparisons. Finally, close collaboration with clinical experts is crucial to identifying potential sources of bias in preprocessing and label interpretation, reducing cognitive bias, and ensuring clinical relevance. Incorporating these practices can significantly improve the transparency, robustness, and translational potential of ML-based diagnostic tools for PD.

## Conclusions

This paper reviews current trends in applying ML technologies in PD diagnosis. In this review, studies are categorised by different data modalities used in the experiments, including neuroimaging, voice, handwriting, gait, and EEG. ML has shown great potential to assist PD diagnosis, and research findings also show that it can be used as a decision-support tool to assist doctors in screening, detecting, and diagnosing PD effectively. Research on applying ML to PD diagnosis still faces many limitations and challenges. We have these issues and proposed several future directions, including the use of explainable AI for model interpretability, data augmentation techniques to generate synthetic data, transfer learning to leverage pre-trained models, federated learning to protect data privacy, and multi-modality approaches to integrate diverse information from different modalities. Herein, a more comprehensive model evaluation, which is beyond traditional metrics such as accuracy and AUC, is essential for ensuring robust, fair, and clinically relevant results. Bias mitigation strategies should also be incorporated to tackle issues such as dataset imbalance, underrepresentation of subgroups, and algorithmic bias. The case studies on five data modalities show that some research papers in this field may face issues with reproduction. Open-source code and reproduced results are essential, and this should be emphasized. Additionally, an ethical framework should be established to ensure these technologies are implemented responsibly and fairly. This comprehensive review aims to reduce the gap between AI experts and medical professionals and help future researchers design ML-based PD diagnosis applications.

## Figures and Tables

**Fig. 14:**
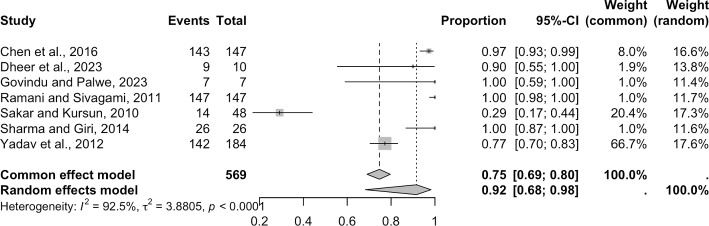
Forest plot for sensitivity across voice modality studies

**Fig. 15:**
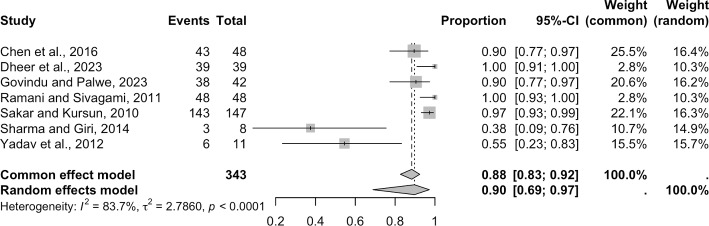
Forest plot for specificity across voice modality studies

## Data Availability

The datasets used in this study are publicly available. The Voice dataset can be accessed at https://archive.ics.uci.edu/dataset/301/parkinson+speech+dataset+with+multiple%20+types+of+sound+recordings, the Gait dataset can be accessed at https://physionet.org/content/gaitpdb/1.0.0/, the EEG dataset can be accessed at https://openneuro.org/datasets/ds002778/versions/1.0.5, the Handwriting dataset can be accessed at https://wwwp.fc.unesp.br/~papa/pub/datasets/Handpd/, and the MRI dataset can be accessed at https://fcon_1000.projects.nitrc.org/indi/retro/parkinsons.html.

## Appendix

We have included a meta-analysis for the voice data modality, which encompasses the effect sizes and relevant statistical trends. For both sensitivity and specificity, we perform the meta-analysis using the meta library in the R language. The forest plots are shown below (Fig. 14, Fig. 15). The forest plots display the variability in sensitivity and specificity across multiple studies of the voice modality.
